# Beyond the *E*-Value: Stratified Statistics for Protein Domain Prediction

**DOI:** 10.1371/journal.pcbi.1004509

**Published:** 2015-11-17

**Authors:** Alejandro Ochoa, John D. Storey, Manuel Llinás, Mona Singh

**Affiliations:** 1 Department of Molecular Biology, Princeton University, Princeton, New Jersey, United States of America; 2 Lewis-Sigler Institute for Integrative Genomics, Princeton University, Princeton, New Jersey, United States of America; 3 Center for Statistics and Machine Learning, Princeton University, Princeton, New Jersey, United States of America; 4 Department of Biochemistry and Molecular Biology, and the Huck Institutes of the Life Sciences, Pennsylvania State University, University Park, Pennsylvania, United States of America; 5 Department of Computer Science, Princeton University, Princeton, New Jersey, United States of America; Microsoft Research, UNITED STATES

## Abstract

*E*-values have been the dominant statistic for protein sequence analysis for the past two decades: from identifying statistically significant local sequence alignments to evaluating matches to hidden Markov models describing protein domain families. Here we formally show that for “stratified” multiple hypothesis testing problems—that is, those in which statistical tests can be partitioned naturally—controlling the local False Discovery Rate (lFDR) per stratum, or partition, yields the most predictions across the data at any given threshold on the FDR or *E*-value over all strata combined. For the important problem of protein domain prediction, a key step in characterizing protein structure, function and evolution, we show that stratifying statistical tests by domain family yields excellent results. We develop the first FDR-estimating algorithms for domain prediction, and evaluate how well thresholds based on *q*-values, *E*-values and lFDRs perform in domain prediction using five complementary approaches for estimating empirical FDRs in this context. We show that stratified *q*-value thresholds substantially outperform *E*-values. Contradicting our theoretical results, *q*-values also outperform lFDRs; however, our tests reveal a small but coherent subset of domain families, biased towards models for specific repetitive patterns, for which weaknesses in random sequence models yield notably inaccurate statistical significance measures. Usage of lFDR thresholds outperform *q*-values for the remaining families, which have as-expected noise, suggesting that further improvements in domain predictions can be achieved with improved modeling of random sequences. Overall, our theoretical and empirical findings suggest that the use of stratified *q*-values and lFDRs could result in improvements in a host of structured multiple hypothesis testing problems arising in bioinformatics, including genome-wide association studies, orthology prediction, and motif scanning.

## Author Summary

Despite decades of research, it remains a challenge to distinguish homologous relationships between proteins from sequence similarities arising due to chance alone. This is an increasingly important problem as sequence database sizes continue to grow, and even today many computational analyses require that the statistics of billions of sequence comparisons be assessed automatically. Here we explore statistical significance evaluation on data that is stratified—that is, naturally partitioned into subsets that may differ in their amount of signal—and find a theoretically optimal criterion for automatically setting thresholds of significance for each stratum. For the task of domain prediction, an important component of efforts to annotate protein sequences and identify remote sequence homologs, we empirically show that our stratified analysis of statistical significance greatly improves upon a combined analysis. Further, we identify weaknesses in the prevailing random sequence model for assessing statistical significance for a small subset of domain families with repetitive sequence patterns and known biological, structural, and evolutionary properties. Our theoretical findings in statistics are relevant not only for identifying protein domains, but for arbitrary stratified problems in genomics and beyond.

This is a *PLOS Computational Biology* Methods paper.

## Introduction

The evaluation of statistical significance is crucial in genome-wide studies, such as detecting differentially-expressed genes in microarray or proteomic studies, performing genome-wide association studies, and uncovering homologous sequences. Different biological applications have settled for different statistics to set thresholds on. In biological sequence analysis, accurate statistics for pairwise alignments and their use in database search [1–3] were introduced with the use of random sequence models and *E*-values two decades ago [4,5]. Sequence similarity searches have evolved further, from the pairwise comparison tools of FASTA [3] and BLAST [5], to sequence-profile [6–8] and profile-profile [9–12] comparisons. While different approaches to detect sequence similarity have relied on a variety of statistics, including bit scores [13,14] and *Z*-scores [3], most modern approaches are based on *E*-values.

Detecting sequence similarity in order to uncover homologous relationships between proteins remains the single most powerful tool for function prediction. Many modern sequence similarity approaches are based on identifying domains, which are fundamental units of protein structure, function, and evolution. Homologous domains are grouped into “families” that may be associated with specific functions and structures, and these domain families organize protein space. Domain families are typically modeled with profile hidden Markov models (HMMs) [13,15]. There are many domain HMM databases, each providing a different focus and organization of domain space, including Pfam [14], Superfamily [16], and Smart [17]. Although HMM-based software, such as the state-of-the-art HMMER program [18], has features that make it superior to its predecessors, accurate significance measures arose only recently [19].

At its core, domain prediction is a multiple hypothesis testing problem, where tens of thousands of homology models (one for each domain) are scored against tens of millions of sequences. Each comparison yields a score *s* and a *p*-value, defined as the probability of obtaining a score equal to or larger than *s* if the null hypothesis holds. While a small *p*-value threshold (for example, 0.05 or smaller) is acceptable to declare a single test significant, this is inappropriate for a large number of tests. Instead, thresholds for domain prediction are typically based on the *E*-value. The *E*-value can be computed from a *p*-value thresholds as *E* = *pN*, where *N* is the number of tests, and yields the expected number of false positives at this *p*-value threshold. *E*-value thresholds make sense for a single database search, especially if few positives are expected. However, *E*-values are less meaningful when millions of positives are obtained, and a relatively larger number of false positives might be tolerated. Moreover, in multiple database query problems, such as BLAST-based orthology prediction [20] or genome-wide domain prediction [21], *E*-values are usually not valid because many searches are performed without the additional multiple hypothesis correction required.

Control of the False Discovery Rate (FDR) is an alternative and appealing approach for multiple hypothesis testing [22]. The FDR is loosely defined as the proportion of all significant tests that are expected to be false, and can be estimated as the *E*-value divided by the number of predictions made. The FDR does not increase with the database size *N* the way the *E*-value does; thus, predictions do not usually lose significance with the FDR as the database grows. The FDR also does not require additional correction in the case of multiple database queries. The FDR is controlled from *p*-values using the Benjamini-Hochberg procedure [22]. The *q*-value statistic is the FDR-analog of the *p*-value, and it provides conservative and powerful FDR control [23]. The *q*-value of a statistic *t* is the minimum FDR incurred by declaring *t* significant [23]. Thus, *q*-values vary monotonically with *p*-values, and they are easily estimated from *p*-values [23]. While *E*-values control the number of false positives, *q*-values control their proportion. The local FDR (lFDR) measures the proportion of false positives in the infinitesimal vicinity of the threshold, and hence it is a “local” version of the FDR [24]; it is also equivalent to the Bayesian posterior probability that a prediction is false [24]. However, *q*-value estimates are much more robust than lFDR estimates, since the former are based on empirical cumulative densities, which converge uniformly to the true cumulative densities [25,26]. On the other hand, lFDR estimates are local fits to the density, so they are comparably more susceptible to noise, especially on the most significant tail of the distribution. The FDR [22], *q*-value [23], and lFDR [24] have all been successfully used in many areas of bioinformatics, including gene expression microarray analysis [24,27,28], genome-wide association studies (GWAS) [27,29], and proteomics analysis [30–34].

Here we introduce the first FDR- and lFDR-estimating algorithms for domain prediction. An essential feature of our approach is that statistical tests are stratified by domain family, rather than pooled. We prove that stratified problems are optimally tackled using the lFDR. For domain prediction, we evaluate how well thresholds based on stratified lFDRs and *q*-values perform using five independent approaches for estimating empirical FDRs. Through extensive benchmarking using the Pfam database and HMMER, we find that using stratified *q*-values increases domain predictions by 6.7% compared to the Standard Pfam thresholds on UniRef50 [35]. In contrast to theory, we also find that *q*-values outperform lFDRs. Further, while the empirical FDRs for most domain families agree with our *q*-value thresholds, some families tend to have larger FDRs; the standard null model appears to be inappropriate for them and yields inaccurate *p*-values. Specifically, families with larger-than-expected empirical FDRs are enriched for those containing repetitive patterns, such as coiled-coils, transmembrane domains, and other low-complexity regions. When only families with as-expected FDRs are considered, the use of *q*-values increases domain predictions by 8.8% compared to the Standard Pfam, and lFDRs further outperform *q*-values, suggesting that further performance improvements are possible if the statistical modeling of repetitive families is improved.

Stratified FDR analyses have been previously explored [36–39], and have been successfully applied to GWAS in particular [29,40,41]. Thus, the same solution we introduce for domain recognition applies to a wide variety of problems in which statistical tests can be analyzed separately, including GWAS (stratifying by candidate or genic regions), orthology prediction (stratifying by each ortholog database search), motif scanning (stratifying by each motif search across a genome), multi-microarray analysis (stratifying by each microarray), and other multi-dataset analyses. Overall, we expect the use of stratified *q*-values and lFDRs to yield improvements in many applications in bioinformatics and beyond.

## Results

### FDR definitions

**Fig 1:**
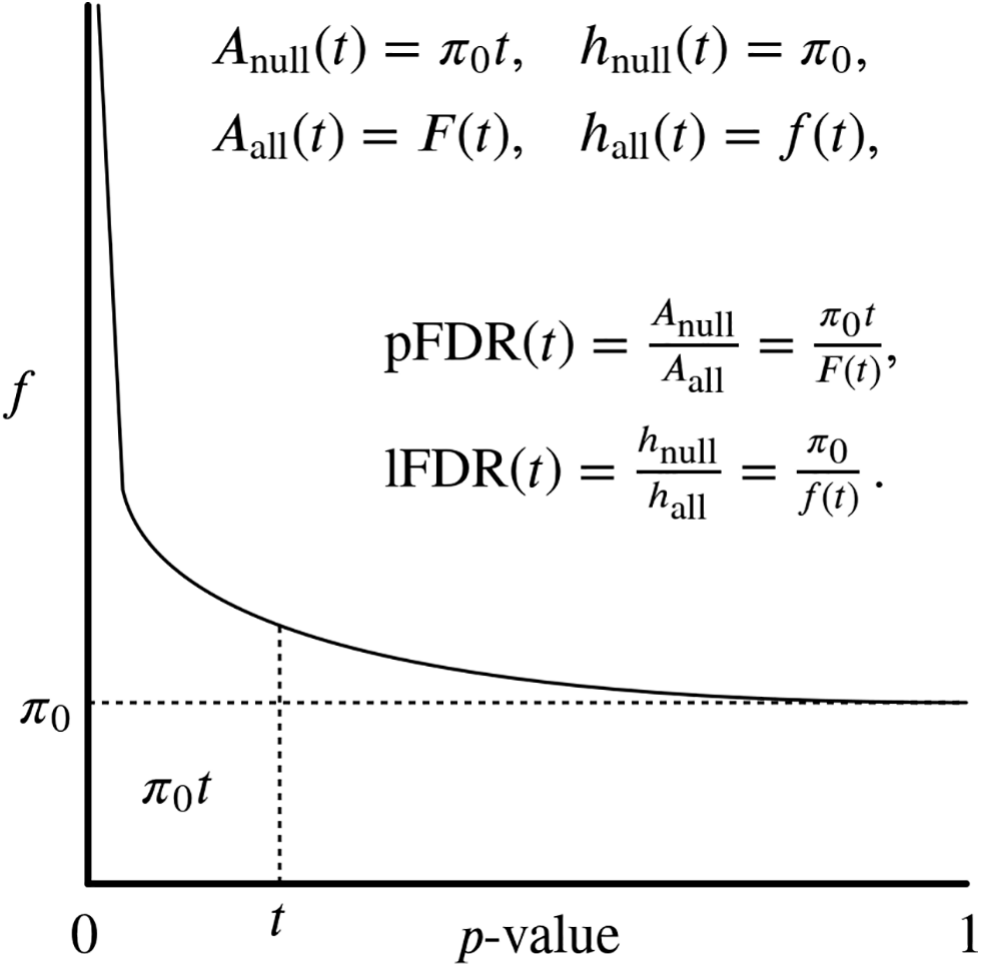
Overview of false discovery rates. Both quantities assume a two-component *p*-value distribution: “null” *p*-values are uniformly distributed (with height *π*_0_ *≤* 1), and “alternative” *p*-values that should peak at *p* = 0. The area of the null component with *p ≤ t* is simply *π*_0_ *t*, while the total area is the cumulative density function *F*(*t*). The total height at *t* is the density function *f*(*t*). The FDR is the proportion of the area with *p ≤ t* that corresponds to the null component. The lFDR parallels the FDR but is a ratio of densities (heights) rather than areas.

We briefly review the relevant FDR definitions; for a comprehensive overview, see [42]. Given a *p*-value threshold *t*, let *V* be the number of false positive predictions, and *R* be the total number of significant tests. Assuming independent *p*-values drawn from a two-component distribution of null and alternative hypotheses (Fig 1), *V* and *R* have expected values of

$$\begin{array}{c} \text{E}\left[V\left(t\right)\right]=t\pi_{0}N,\\ \text{E}\left[R\left(t\right)\right]=F\left(t\right)N, \end{array}$$

where π_0_ is the proportion of tests which are truly null, *N* is the total number of tests, and *F*(*t*) is the cumulative density of *p*-values [23,24,43]. Note that E[*V*(*t*)] gives the *E*-value.

There are two closely-related versions of the FDR used in our work: the positive FDR (pFDR) and marginal FDR (mFDR) [42,43], defined as

$$\begin{array}{c} \text{pFDR}=E\left[\frac{V}{R}|R>0\right],\\ \text{mFDR}=\frac{E\left[V\right]}{E\left[R\right]}. \end{array}$$

The advantages of the pFDR compared to the original FDR definition of Benjamini and Hochberg [22] are discussed in [43]. If *p*-values are drawn independently from the two-component distribution of Fig 1, the pFDR and mFDR were proven to be equivalent to the following posterior probability [43]:

$$\text{pFDR}\left(t\right)=\text{mFDR}\left(t\right)=\text{Pr}\left(H=0|p\leq t\right)=\frac{t\pi_{0}}{F\left(t\right)},$$

where *H* = 0 denotes that the null hypothesis holds. This quantity is sometimes called the “Bayesian FDR” [24]. The pFDR and mFDR are also asymptotically equal under certain forms of “weak dependence,” as defined in [44]. Our domain prediction problem has large sample sizes and weak dependence: our dataset contains millions of protein sequences and thousands of HMMs, and null *p*-values are only dependent for very similar sequences and similar HMMs. Dependent tests represent a very small subset of all hypotheses tested, even on each stratum (for any one HMM). For this reason, we use FDR to refer loosely to all these FDR definitions.

The local FDR (lFDR) is the Bayesian posterior error probability defined as [24]

$$\text{lFDR}\left(t\right)=\text{Pr}\left(H=0|p=t\right)=\frac{\pi_{0}}{f\left(t\right)}\;,$$

where *f*(*t*) = *F*'(*t*) is the *p*-value density at *t*. Thus, while the pFDR is a ratio of areas, the lFDR is a ratio of densities (Fig 1) [45].

The *q*-value of a statistic *t* is the minimum pFDR incurred by declaring *t* significant [23]. Estimated *q*-values are efficiently constructed from *p*-values, and conservatively estimate the pFDR [23]. Specifically, *q*-value and lFDR estimation are based on the above formulas, where π_0,_ *F*(*t*) and *f*(*t*) are replaced by estimates. See the Supp. Methods in S1 Text for the algorithms for estimating *q*-values and lFDRs.

### Equal stratified lFDR thresholds maximize predictions while controlling the combined FDR

Here we prove that the lFDR gives optimal thresholds for stratified problems. For domain prediction, each domain family defines a stratum. We wish to find *p*-value thresholds *t*_*i*_ per stratum *i* that maximize the number of predictions across strata while constraining the maximum FDR of the strata combined. Optimality of the lFDR here is consistent with the related Bayesian classification problem, where posterior error probabilities are also optimal [43].

Let the FDR model quantities *N*_*i*_, *π*_0,i_, *F*_*i*_(*t*_*i*_) and *f*_*i*_(*t*_*i*_) be given per stratum *i*. We desire to maximize the expected number of predictions across strata

$$\sum_{i}F_{i}\left(t_{i}\right)N_{i}\;,$$

while constraining the “combined” FDR, which we define as the sum of expected false positives across strata divided by the total number of expected predictions, to a maximum value of *Q*, or

$$\frac{\sum_{i}t_{i}\pi_{0,i}N_{i}}{\sum_{i}F_{i}\left(t_{i}\right)N_{i}}\leq Q\;.$$

This problem is solved using the Lagrangian multiplier function *Λ*, with the constraint set to strict equality, in a formulation that avoids quotients:

$$\begin{array}{l} \Lambda =\sum_{i}F_{i}\left(t_{i}\right)N_{i}+\lambda \left(\sum_{i}t_{i}\pi_{0,i}N_{i}-Q\sum_{i}F_{i}\left(t_{i}\right)N_{i}\right)\\ \;=\sum_{i}F_{i}\left(t_{i}\right)N_{i}\left(1-\lambda Q\right)+\lambda t_{i}\pi_{0,i}N_{i}. \end{array}$$

Taking the partial derivative of *Λ* with respect to *t*_*j*_, we obtain a necessary condition for optimality,

$$\begin{array}{c} \frac{\partial \Lambda }{\partial t_{j}}=f_{j}\left(t_{j}\right)N_{j}\left(1-\lambda Q\right)+\lambda \pi_{0,j}N_{j}=0\;\Leftrightarrow \\ Q-\frac{1}{\lambda }=\frac{\pi_{0,j}}{f_{j}\left(t_{j}\right)}={\text{lFDR}}_{j}\left(t_{j}\right), \end{array}$$

which shows that the lFDR of each stratum must be equal, since the last equation has the same value for every *j*. Optimality of the lFDR also holds when constraining the combined *E*-value instead of the combined FDR (Supp. Methods in S1 Text).

### Obtaining *E*-values, *q*-values, and lFDRs for domains

Each of the 12,273 Pfam domain families was used to scan for domains in each of 3.8 million proteins of UniRef50 (Supp. Methods in S1 Text), resulting in a total of 47 billion tests. Domain predictions are stratified by family (HMM), and each stratum contains *p*-values from which we estimate *q*-values and lFDRs. We note that standard *q*-value and lFDR implementations fail for domain data for two reasons. First, modern HMM software only reports the smallest *p*-values due to heuristic filters [19]. Second, homologous families (grouped into “superfamilies” [16] or “clans” [14]) produce frequent overlaps that are resolved by removal of all but the most significant match, and thus there are fewer predictions than an independent family analysis would predict, which leads to underestimated FDRs. To address these issues, we remove overlapping domains (keeping those with the smallest *p*-values), and then estimate *q*-values and lFDRs with methods adapted for censored *p*-values (Methods). For comparison, we also use *E*-value thresholds and the “Standard Pfam” curated bitscore thresholds (also called “Gathering” or “GA” [14]). Note that a stratified *E*-values approach (separating families) is no different from a combined *E*-value approach in that the ranking of predictions is preserved, since the number of proteins, or tests, is the same per stratum; the stratified *E*-value threshold equals the combined *E*-value threshold divided by the number of strata. Similarly, a combined *q*-value or lFDR approach (obtained by combining the *p*-values of all strata) also preserves the *E*-value rankings.

### Empirical FDR tests

We estimate the true FDR via “empirical” FDR tests, to compare all methods on an equal footing, but also to test the accuracy of *q*-value estimates. We created or adapted five tests, each of which labels domain predictions as either true or false positives (TP, FP) using different statistical and biological criteria. The proportion of predictions labeled FP estimates the FDR.

**Fig 2:**
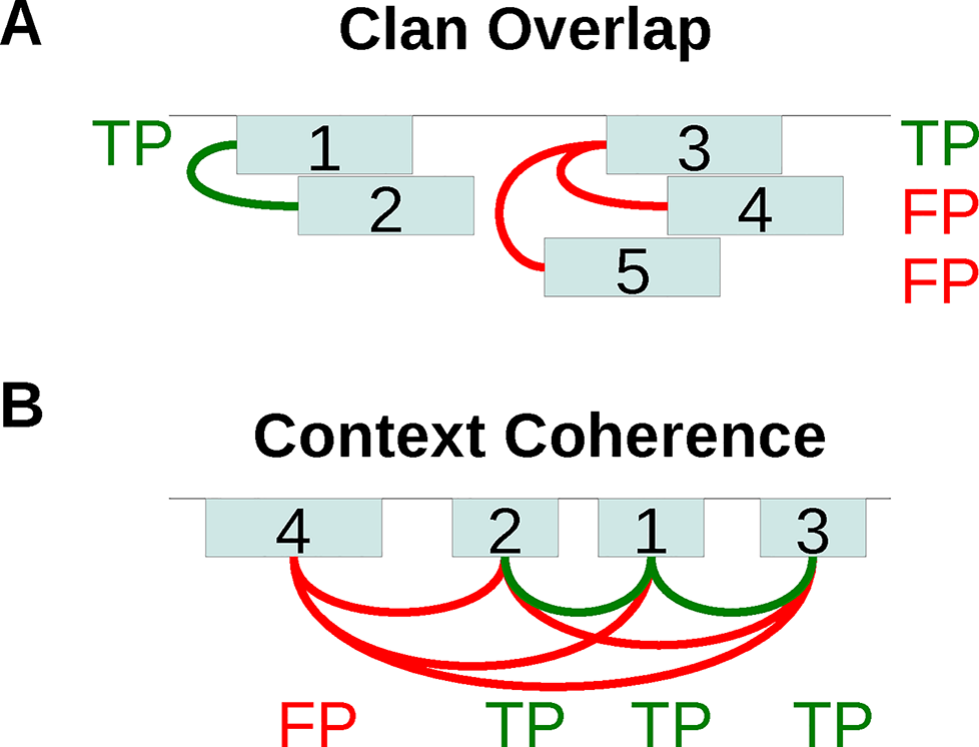
Illustration of the empirical FDR tests ClanOv and ContextC. Both tests rank domain predictions (teal boxes) by *p*-value (numbers within boxes are ranks). (**A)** In ClanOv (“Clan Overlap”), highest-ranking domains are considered as TPs, domains that overlap higher-ranking domains of the same clan (green connections) are removed (not counted toward the FDR or downstream overlaps), and domains that overlap higher-ranking domains of different clans (red connections) are considered FPs. (**B)** In ContextC (“Context Coherence”), the highest-ranking domain prediction in a sequence is considered a TP. Subsequent domains are considered TPs if there is at least one higher-ranking domain such that their families have been observed together before in UniProt (green connections), and otherwise they are considered FPs (all red connections).

For simplicity, only two tests are described here in detail and are featured in the main figures. First, the ClanOv (“Clan Overlap”) test is based on the expectation that overlapping domain predictions should be evolutionarily related [46]. Pfam annotates related families via clans. In this test, domain predictions are ranked by *p*-value, highest ranking domains are considered as TPs, domains that overlap a higher-ranking domain of the same clan are removed (since they would not be counted as separate predictions), and domains that overlap a higher-ranking domain of a different clan are considered FPs (Methods, Fig 2A). All FPs in this test would not be predicted by our method when overlaps are removed; nevertheless, this method estimates well the amount of noise. Second, the ContextC (“Context Coherence”) test is based on whether domain pairs predicted within a sequence have been observed together before [47]. For each sequence, domain predictions are ranked by *p*-value, and the highest ranking domain is always a TP. Subsequently, a domain is a TP if its family has previously been observed with the family of at least one higher-ranking domain, and otherwise it is a FP (Methods, Fig 2B).

The principles behind the other three tests are described here briefly: OrthoC (“Ortholog Set Coherence”) is based on the expectation that orthologous proteins contain similar domains [48], RevSeq (“Reverse Sequence”) estimates noise based on domains predicted on reversed amino acid sequences [49], and MarkovR (“Markov Random”) estimates noise based on domains predicted on random sequences generated from a second-order Markov model (Supp. Methods and Fig A in S1 Text).

**Fig 3:**
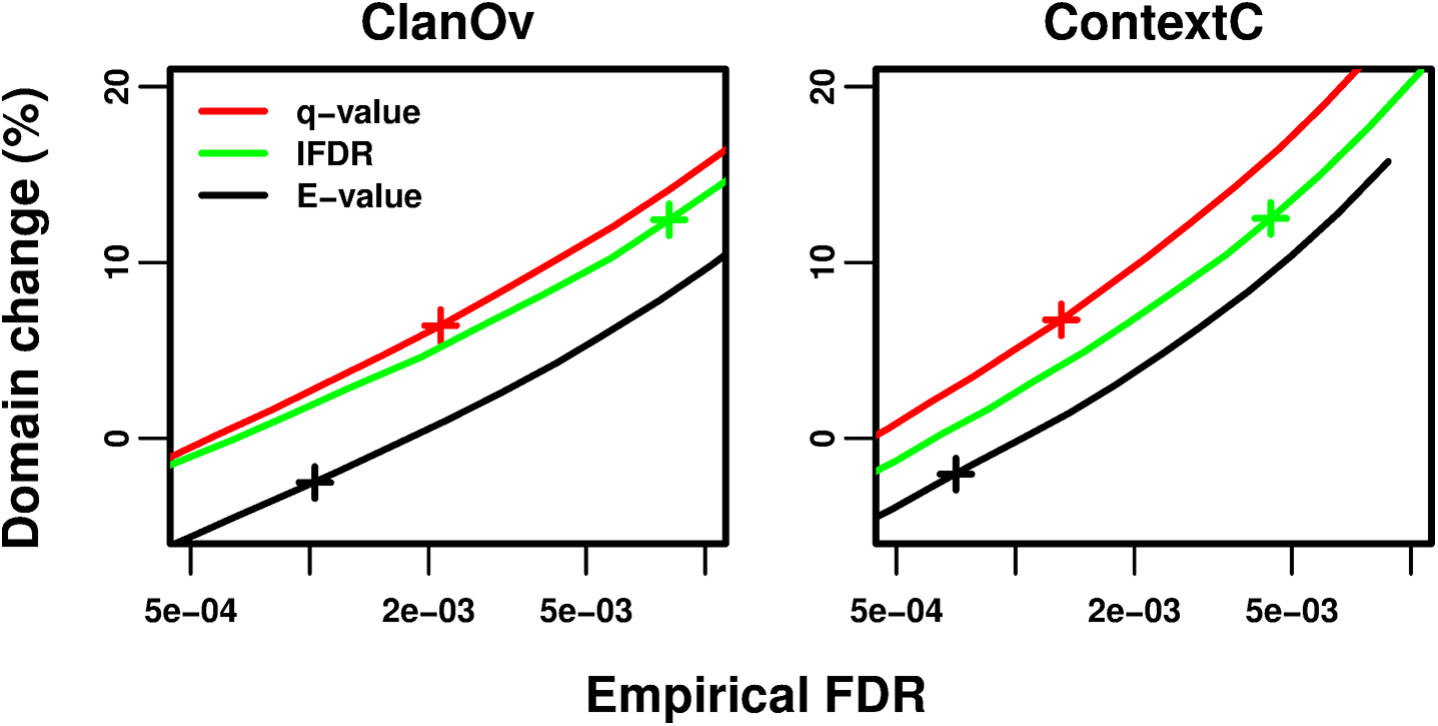
Change in domain predictions while controlling empirical FDRs. In each panel, a different empirical FDR test (*x*-axis, note log scale) is used to evaluate each method at a series of thresholds. The number of domain predictions is turned into a percent change relative to the number of Standard Pfam predictions (*y*-axis). Curves correspond to: *E*-values (black), cross marks p *≤* 1.3e-8; stratified *q*-values (red), cross marks *q ≤* 4e-4; stratified lFDRs (green), cross marks lFDR *≤* 2.5e-2. The *q*-value and lFDR thresholds marked with crosses correspond to the median across domain families of the Standard Pfam thresholds mapped theoretically to those statistics (Supp. Results in S1 Text). All curves have standard error bars in both dimensions, which are not always visible. Standard Pfam is not plotted as both the ClanOv and ContextC tests are based on Standard Pfam predictions.

Methods are compared at the same empirical FDR based on the number of domain predictions (Fig 3 and Fig B in S1 Text), unique families per protein (Fig C in S1 Text), amino acids covered (Fig D in S1 Text), and proteins with predictions (Fig E in S1 Text), as well as their total “GO information content” scores (derived from the Gene Ontology [50] and MultiPfam2GO [51]; Supp. Methods and Fig F in S1 Text).

### Stratified *q*-values predict more domains than the Standard Pfam, *E*-values, and lFDRs

Stratified *q*-value thresholds outperform *E*-values in all tests (Fig 3, Fig B in S1 Text). While stratified lFDR thresholds are superior to *E*-values in all tests, they are unexpectedly outperformed by *q*-values on most tests. We hypothesize that lFDR estimates are less robust than *q*-values due to errors in *p*-values; these errors most likely arise because of weaknesses in the standard null model. The Standard Pfam is not evaluated using ClanOv and ContextC (Fig 3); these tests are based on the Pfam clans and observed domain pairs, so the Standard Pfam has zero empirical FDRs in both. However, *q*-values outperform the Standard Pfam in two of the three fair tests (OrthoC, MarkovR) and perform similarly in RevSeq (Fig B in S1 Text). The same trends hold if the combined empirical *E*-value is controlled (Supp. Methods and Fig G in S1 Text).

### *Q*-value predictions are more informative than those of Standard Pfam, dPUC

**Fig 4:**
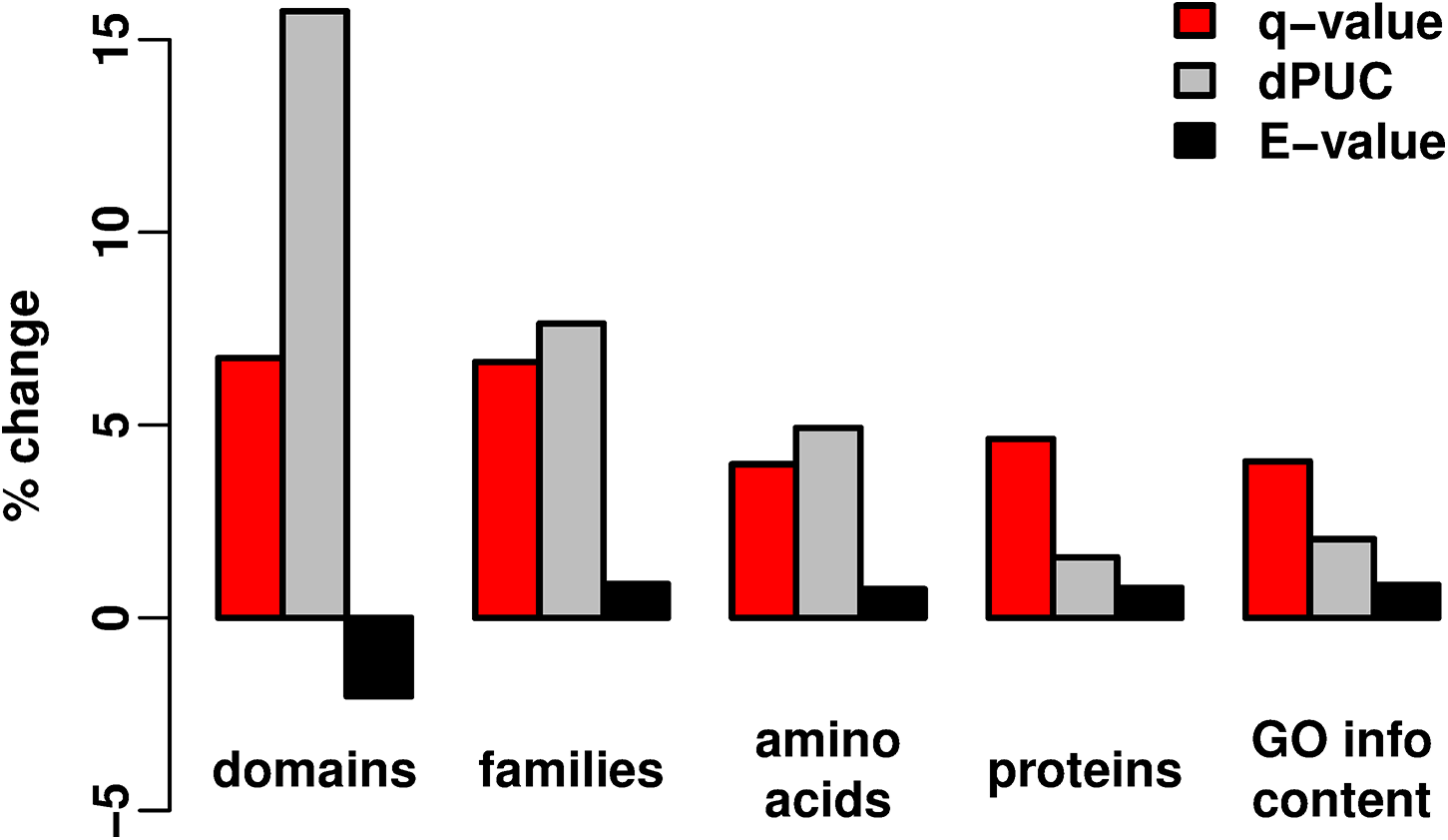
Percent changes for several metrics relative to the Standard Pfam. We count “domain” predictions in UniRef50; unique “families” per protein over all proteins; “amino acids” covered by domains over all proteins, without double-counting amino acids covered by multiple domains; “protein” counts with any predicted domain; and “GO info content”, which sums the functional information content of all proteins with domain predictions (Supp. Methods in S1 Text). All quantities are turned into percent changes relative to the respective numbers from the Standard Pfam. *Q*-value uses *q ≤* 4e-4, *E*-value uses *p ≤* 1.3e-8, and dPUC uses a “candidate domain *p*-value threshold” of 1e-4, which gives comparable empirical FDRs as the Standard Pfam.

We measure improvements not only of domain counts, which may be inflated for families with many small repeating units, but also of unique family counts. We also measure the information content based on the GO terms associated with domain predictions [51] (Supp. Methods in S1 Text). To have amounts of noise comparable to Pfam, we calculate *p*- and *q*-value equivalents to the Standard Pam thresholds for each family (Supp. Results in S1 Text). The medians of these distributions give thresholds of *q* ≤ 4e-4, and for *E*-values, *p* ≤ 1.3e-8 (Supp. Results in S1 Text). *Q*-values improve all metrics consistently relative to the Standard Pfam (between 4–7%, Fig 4). *E*-values predict 2% fewer domains than the Standard Pfam, but slightly outperform Pfam in the other metrics (Fig 4).

We also evaluated dPUC, a prediction method based on domain context [48,52]. dPUC also improves upon the Standard Pfam in all cases (Fig 4). dPUC increases domains more than *q*-values, but their unique family count and amino acid coverage are comparable, and *q*-values best dPUC for protein counts and GO information content. This is because dPUC predicts more repeat domains (of the same family) and tends to restrict new predictions to proteins that already had Standard Pfam predictions. In contrast, *q*-values increase domains at the same rate as they increase protein coverage, which increases information the most. Thus, while stratified *q*-values predict fewer domains than dPUC, those domains tend to be more informative than the dPUC predictions at comparable FDRs.

### Empirical FDRs and *q*-values disagree in few domain families

**Fig 5:**
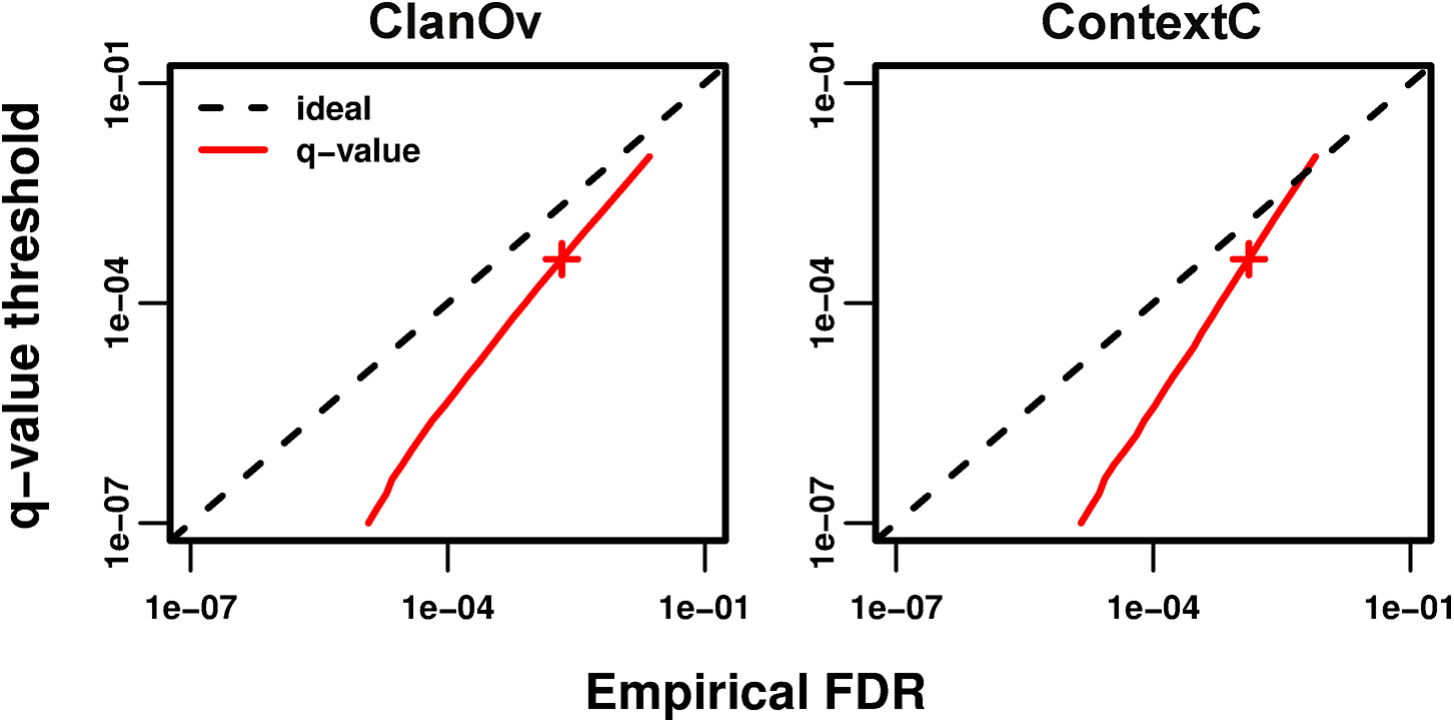
Comparison of *q*-value thresholds and empirical FDRs. In each panel, for each *q*-value threshold, observed empirical FDRs are computed (by ClanOv, left, and ContextC, right) and the relationship between these two quantities is shown in red. Since *q*-values control FDRs when input *p*-values are correct, ideally these data fall on the *y* = *x* line (dashed black lines). Values below the dashed line correspond to empirical FDRs that are larger than *q*-values. Smaller FDRs correspond to more stringent predictions and therefore include fewer predictions. All *x* and *y*-axes have the same range for ease of comparison and are in log scale. The red cross marks *q ≤* 4e-4.

**Fig 6:**
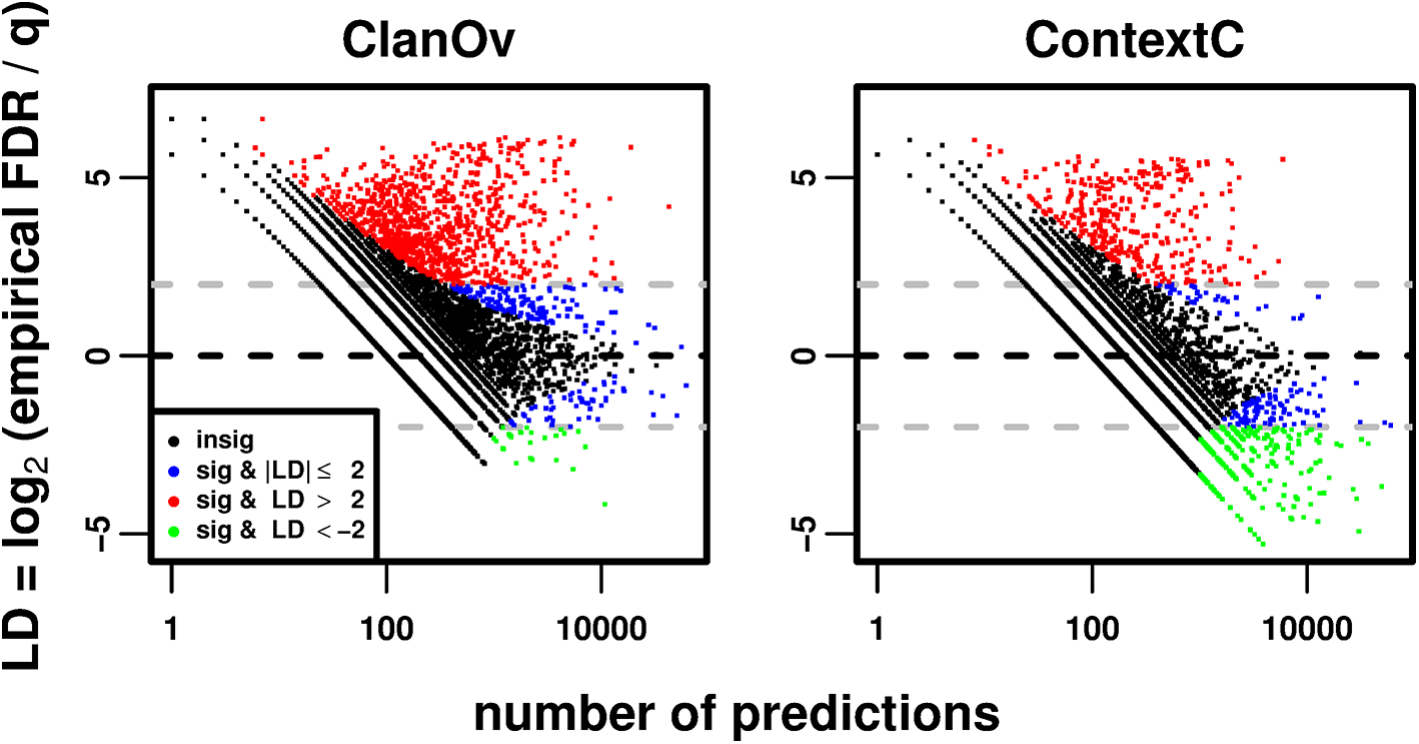
Identification of domain families with empirical FDRs that differ significantly from expectation. In each panel, the empirical FDR is computed (by ClanOv, left, and ContextC, right) for each domain family at *q ≤* 1e-2, and the log-deviation (LD) of this empirical FDR from the threshold is plotted on the *y*-axis relative to the number of predictions at this threshold (*x*-axis). Zero LD corresponds to perfect agreement, while positive and negative numbers correspond to underestimated and overestimated empirical FDRs, respectively. The LD values of 0, 2, and -2 are marked with horizontal black, gray, and gray dashed lines respectively. Families are plotted as black dots if their deviations are insignificant via a Poisson test (Methods), blue if the deviations are significant but the effect size is small (|LD| *≤* 2), red if the deviations are significant and have a large positive effect size (LD > 2), and green if the deviations are significant and have a large negative effect size (LD < -2).

We find large disagreements between *q*-values and our empirical FDRs tests (except for MarkovR; Fig 5, Fig H in S1 Text). Interestingly, the disagreement is proportionally larger for smaller FDRs, and shrinks as the FDR grows (Fig 5). We hypothesize that a few families are too noisy at stringent thresholds, and this subset becomes proportionally smaller as all families are allowed greater noise. To test this, we compute empirical FDRs separately per family at a threshold of *q ≤* 1e-2 (Methods). This threshold gives a greater FDR than the Standard Pfam (Supp. Results in S1 Text), which is desirable here as many families have few predictions at more stringent thresholds. Since large deviations between the empirical FDRs and *q*-values may arise due to low sampling, significance is assessed by modeling this random sampling (Methods). We find that most families (92–99%, Table A in S1 Text) have FDRs close to the *q*-value threshold or have statistically insignificant differences (blue and black data in Fig 6, Fig I in S1 Text).

### Empirical FDRs elevated in families with repetitive patterns

**Fig 7:**
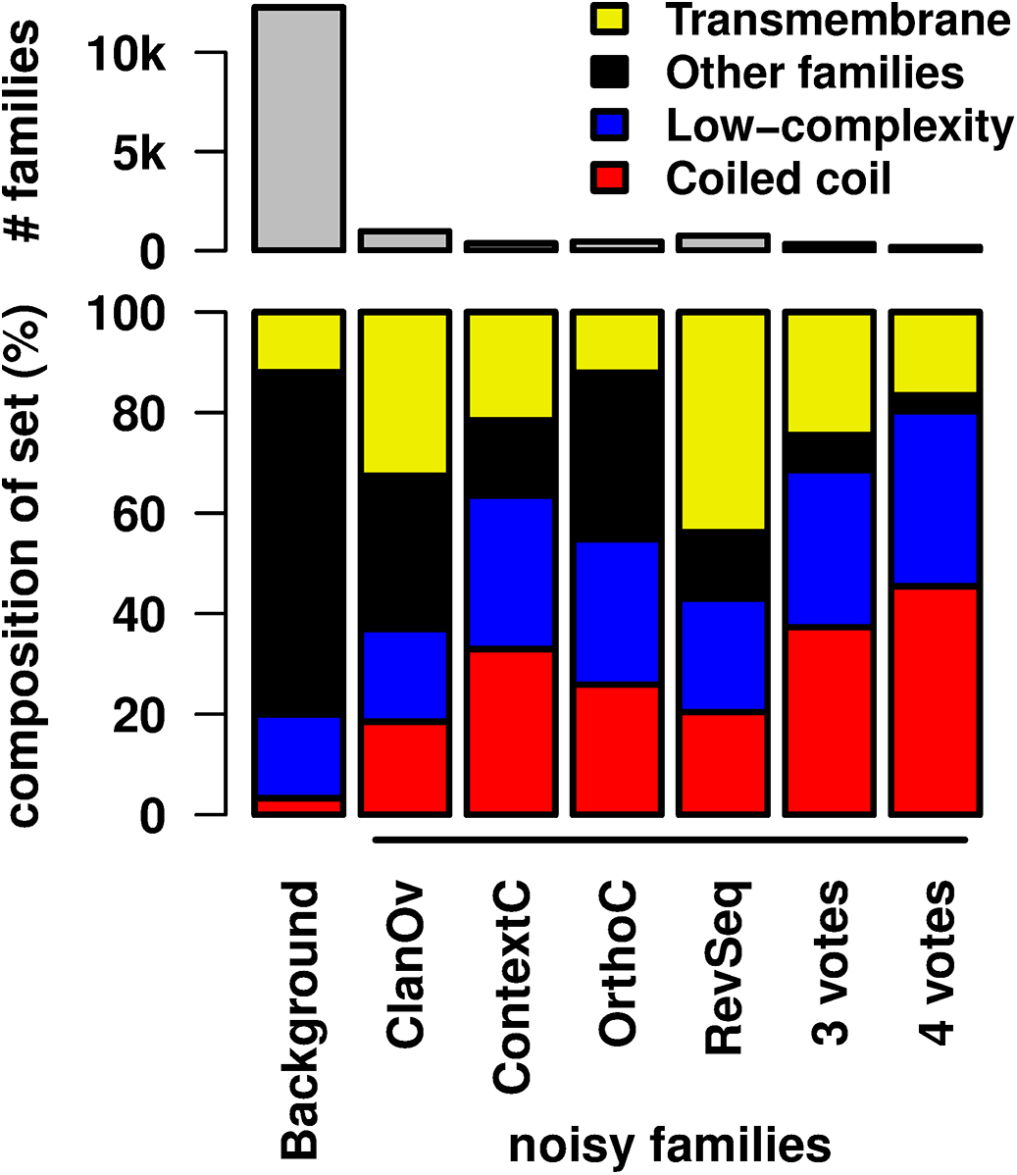
Families with increased noise are enriched for repetitive patterns. Each Pfam family is classified as transmembrane domain, low-complexity region, coiled-coil, or “other” (Methods). The “background” bars correspond to all Pfam families, and the other bars correspond to noisy families, or those with significantly larger empirical FDRs than expected with *q ≤* 1e-2 using either ClanOv, ContextC, OrthoC, RevSeq, at least three of these tests (3 votes), or all four tests (4 votes). The top (gray) bars show the set size, and the bottom bars (colors) show the set composition. Category enrichments are evaluated using the hypergeometric distribution, and two-sided *p*-values with *p ≤* 0.01 are declared significant. All noisy sets are significantly enriched for coiled coils and de-enriched for “other” families. Low-complexity regions are significantly enriched in all sets except ClanOv. Transmembrane domains are significantly enriched in all sets except OrthoC and “4 votes.”

Four tests (ClanOv, ContextC, OrthoC, and RevSeq) detect many families with significantly larger FDRs than expected (3–8%, Table A in S1 Text). These families are significantly enriched for those containing coiled-coils, transmembrane domains, and low-complexity regions (Fig 7; Methods). There are fewer families with significantly smaller FDRs than expected (0–2%, Table A in S1 Text), and they do not appear to share common patterns. Only the MarkovR test conforms to expectation, with no families having significantly larger FDRs than expected and 0.1% of families having significantly smaller FDRs than expected.

### Assigning domain families to noise classes

We use the four tests (excluding MarkovR) to assign families into mutually-exclusive classes by majority rule. The “increased-noise” families have significantly large positive deviations (see Methods; red in Fig 6) in at least three tests. The “decreased-noise” families have significantly large negative deviations (green in Fig 6) in at least three tests. Lastly, the families with “as-expected-noise” have small deviations (blue and some black in Fig 6) in at least three tests. There are 327 increased-noise families (2.7% of Pfam, S1 File), one decreased-noise family (HemolysinCabind), and 4433 as-expected-noise families (36%, S2 File). There are 7512 unclassified families in Pfam (61%). Using these classes, we find that the Standard Pfam has more stringent thresholds (in terms of *q*-values) for increased-noise as compared to as-expected-noise families, but many increased-noise family thresholds remain too permissive (Supp. Results and Fig J in S1 Text).

### The lFDR outperforms *q*-values in families with as-expected noise

**Fig 8:**
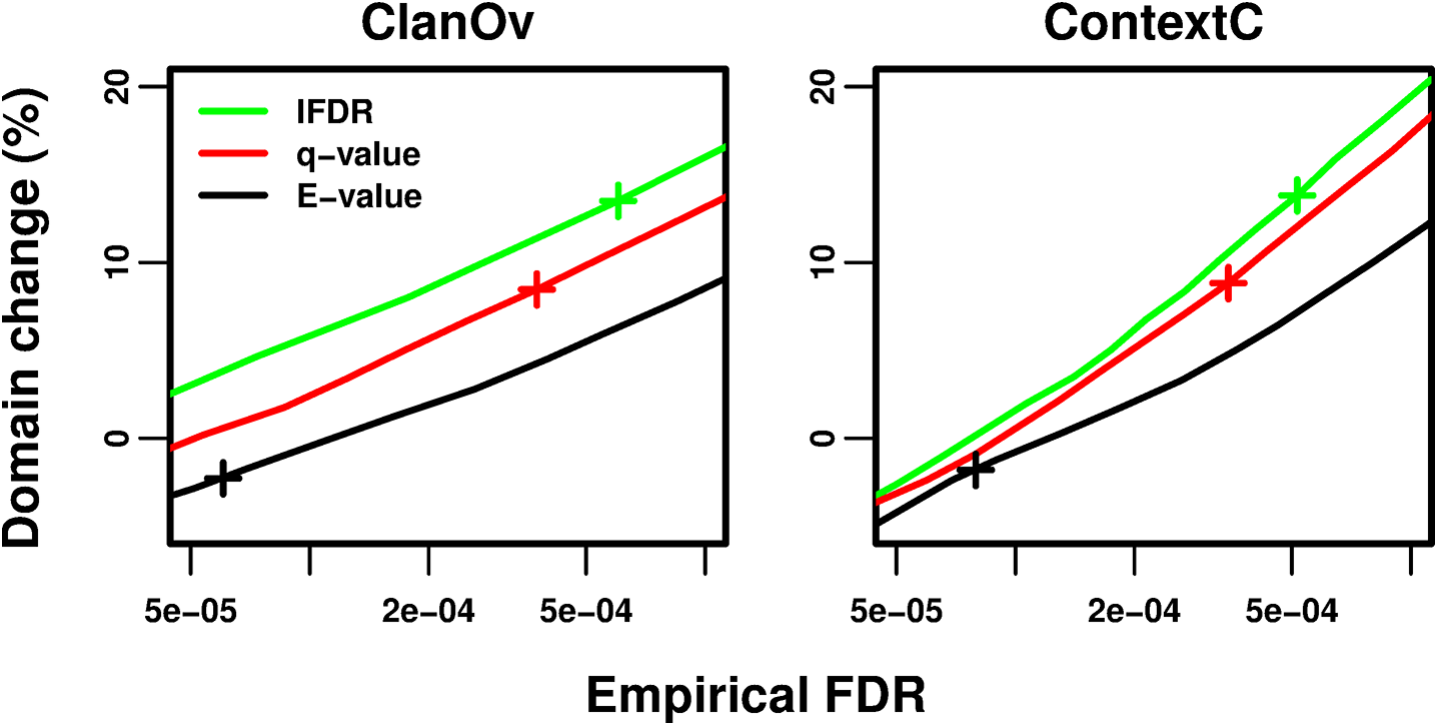
Domain change while controlling empirical FDRs, restricted to families with as-expected noise. This figure is exactly like Fig 3 except that only families with “as-expected noise” are used in the benchmarks. See Fig 3.

Empirical FDRs agree more with *q*-values in as-expected-noise families than in all families combined, although some disagreement remains (Fig K in S1 Text). In these families, lFDRs outperform *q*-values (Fig 8), as we expect from our theoretical results when the underlying *p*-values are correct. Compared to the Standard Pfam, domain counts at *q ≤* 4e-4 increase from 6.7% in all families to 8.8% in as-expected noise families (similar increases are observed on all metrics; Fig L in S1 Text), and lFDRs further improve upon *q*-values. Thus, lFDRs may become more useful should *p*-values for all families improve in the future.

### Tiered stratified *q*-values

The previous methods describe a single “domain” threshold set via the stratified *q*-value or lFDR analysis. However, HMMER provides additional information in the form of “sequence” *p*-values, which score the presence of domain families combining the evidence of repeating domains. Only 2.3% families have different sequence and domain Standard Pfam thresholds [14]. Here we define “two-tier” thresholds using the FDR. In the first tier, we compute *q*-values from the sequence *p*-values and set the threshold *Q*_*seq*_. In the second tier, we compute *q*-values on the domain *p*-values, only for the domains in sequences that satisfied the sequence threshold, and set the threshold *Q*_*dom|seq*_ (corresponding to a FDR conditional on the first threshold). The final FDR is approximately *Q*_*seq*_ *+Q*_*dom|seq*_ if both thresholds are small and under an independence assumption (Supp. Methods in S1 Text). For simplicity, we only evaluate the case where *Q*_*seq*_ = *Q*_*dom|seq*_.

Tiered *q*-values predict many more domains, at any fixed empirical FDR, than domain *q*-values and domain lFDRs, our previous two best statistics, consistently and by very large margins (Fig B in S1 Text). Tiered *q*-values also outperform other methods in predicting new families per sequence (Fig C in S1 Text); the entire signal of these families comes from combining repeating units, none of which is significant by itself. There is also a large increase in amino acid coverage (Fig D in S1 Text), and a smaller increase in protein coverage (Fig E in S1 Text) and GO information content (Fig F in S1 Text). Tiered *q*-values also compare favorably to dPUC [48], matching the superior domain improvements of dPUC, and outperforming dPUC in all other metrics (Fig M in S1 Text). Thus, tiered *q*-values retain the strengths of domain *q*-values while powerfully leveraging the limited context information of repeating domains present in sequence *q*-values. However, the estimated FDRs of tiered *q*-values are less accurate than for domain *q*-values (Fig H in S1 Text), and remain less accurate in as-expected-noise families (Fig K in S1 Text). For this reason, tiered stratified *q*-values are experimental: although they are more powerful than domain-only *q*-values, they do not, as described, control the FDR as well.

## Discussion

In multiple hypothesis testing, the FDR and lFDR are straightforward approaches for controlling the proportion of false positives and the posterior error probability, respectively. The *q*-value is a statistic for controlling the FDR that is less biased and more flexible than previous FDR procedures such as the one from Benjamini and Hochberg [22]. Benchmarks based on empirical FDRs have been a part of recent works studying protein and DNA homology [47,48,52,53]; however, those approaches have used expensive simulations rather than estimating FDRs directly from *p*-*v*alues (or *E*-*v*alues), as *q*-*v*alues do very efficiently. Our work is, to the best of our knowledge, the first attempt at applying *q*-*v*alues and lFDRs to domain identification, thus advancing the statistics of this field.

Our theoretical work revealed that the lFDR, which is the Bayesian posterior probability that a prediction is false, is the optimal quantity to control in stratified problems. Stratified lFDR control has previously been found to optimize stratified thresholds in the related problem of minimizing the combined false non-discovery rate while controlling the combined FDR [37]. The lFDR also arises naturally in Bayesian classification problems [43]. Stratified lFDR thresholds ensure the least confident predictions of each stratum have the same posterior error probability. However, we found that estimated *q*-values are more robust than our lFDR estimates for domain predictions, where the underlying *p*-value estimates are imperfect [45] (Fig 3).

We extended the domain stratified *q*-value approach into what we call tiered stratified *q*-values, by setting *q*-value thresholds on both the sequence and domain statistics reported by HMMER. While accurate FDR estimation of this procedure remains a challenge, tiered *q*-values successfully leverage the additional signal of repeating domains to increase predictions (Fig M in S1 Text). There are other successful approaches, such as dPUC [48] and CODD [52], that use the broader concept of domain context (or co-occurrence) to improve domain predictions. Remarkably, tiered *q*-values perform as well or better than as dPUC under all metrics (Fig M in S1 Text), even though tiered *q*-values only utilize the context signal of repeating domains, while dPUC additionally considers context between families [48]. In the future, tiered *q*-values could be combined with dPUC to yield further improvements in domain prediction.

We introduced a suite of empirical FDR tests to evaluate domain predictions. Altogether, these tests are powerful means for evaluating the correctness of predictions (“Evaluation of empirical FDR tests” Supp. Results in S1 Text). Four of our tests consistently revealed flaws in the estimates of statistical significance for some families. We found a strong enrichment among noisy families for coiled coils, transmembrane domains, and other low-complexity regions. These problematic domain categories have been noted elsewhere [46,54,55], and *ad hoc* solutions have been proposed [54,56]. However, none of these solutions are implemented by standard software such as BLAST and HMMER [56]. In our view, obtaining correct statistics for these repetitive families should be the top priority of the field of sequence homology. Nevertheless, most families in Pfam appear to have correct statistics, and the advantage of using *q*-values and lFDRs is clear. In the future, the standard sequence similarity software packages should be able to report these stratified statistics natively rather than as a post-processing step as is done here.

Domain prediction is one case where stratified FDR and lFDR control are desirable, since domain families occur with vastly different frequencies and are thus associated with differing amounts of true signal. However, the same holds for other applications, such as BLAST-based orthology prediction [20], since some ortholog groups are orders of magnitude larger than others. FDR and lFDR control may also improve iterative profile database searches, such as PSI-BLAST [6], as well as numerous other sequence analysis problems.

The basis of our work is a general theorem applicable to naturally stratified statistical tests. Whether the combined FDR or *E*-value is constrained, equal stratified lFDR thresholds are required to maximize predictions. Besides limits on sample size, the strata may be arbitrary, so our result can be broadly applied to multiple hypothesis testing problems. In motif scanning, for example *in silico* transcription factor (TF) binding site identification, the position weight matrix of each TF may yield a *p*-value per match [57], and the number of binding sites per TF may vary by orders of magnitude across different TFs. Here, we recommend computing lFDRs stratified by TF, and setting equal lFDR thresholds across TFs. For protein domains, one could further stratify *p*-values using taxonomy, since domain family abundances vary greatly across the kingdoms of life (archaea, bacteria, eukarya, and viruses) [58,59]. In sum, we have demonstrated the practical utility of our theoretical contributions to domain prediction, which are likely to influence many applications in bioinformatics and beyond.

## Methods

### HMMER *p*-values

A *p*-value distribution is required to estimate *q*-values and lFDRs. HMMER reports two kinds of *p*-values. The “sequence” *p*-value combines every domain of the same family on a protein sequence, while the “domain” *p*-value is limited to each domain instance. The sequence *p*-value thus reports whether the protein sequence as a whole contains similarity to the HMM, whereas the domain *p*-value scores individual domain units within the sequence. We obtained domain predictions with *p*-values on UniRef50 [35] and OrthoMCL5 [20] proteins using hmmsearch from HMMER 3.0 and HMMs from Pfam 25 with these parameters: the heuristic filters “--F1 1e-1--F2 1e-1--F3 1e-2” allow sequence predictions with “stage 1/2/3” *p*-value thresholds of 0.1, 0.1, and 0.01, respectively. Moreover, we obtain *p*-values using “-Z 1--domZ 1”. Lastly, we remove domains with *p*>0.01 by adding “-E 1e-2--domE 1e-2”.

### Overview of *q*-value and lFDR estimation for domains

For each domain family HMM, we use its HMMER *p*-values over a protein database to estimate *q*-values and lFDRs. We use standard methods [27,45] adapted for censored tests since HMMER3 only reports the most significant *p*-values while standard methods require all *p*-values. Notably, HMMER3 does not provide complete *p*-values even if filters are removed [60], and only small *p*-values are accurate [19], so the full set of *p*-values is not useful. Moreover, the filters are desirable to reduce HMMER3's runtime. The Supp. Methods (S1 Text) reviews these standard methods for estimating *q*-values and lFDRs, and details our adaptations for domains. Briefly, we remove overlaps between domain predictions ranking by *p*-value, before computing *q*-values and lFDRs; otherwise, the amount of true positive may be overestimated because overlapping domains will be counted double, a common case within Pfam clans. Secondly, the standard approaches require all *p*-values solely to estimate *π*_0_, here roughly the proportion of proteins that do not contain a domain family. We set *π*_0_ = 1, which gives slightly more conservative *q*-values and lFDRs than otherwise. Our software for computing stratified *q*-values, lFDR estimates and tiered *q*-values from HMMER3 is DomStratStats 1.03, available at https://github.com/alexviiia/DomStratStats.

### Baseline threshold methods

We compare new and standard domain prediction approaches over a range of relevant empirical FDRs. We vary thresholds based on stratified *q*-values and lFDRs, and compare their performances to thresholds varied by *E*-values and extensions of the Standard Pfam. Stratified domain *E*-values are computed from the HMMER *p*-values by multiplying them by the number of proteins in UniRef50, as hmmsearch would compute them. The “Standard Pfam” has two expert-curated thresholds per family, for domain and sequence bitscores respectively (Pfam calls them “gathering” thresholds) [14]. For all methods, domain overlaps are removed ranking by *p*-value. Overlaps between families in the “nesting” list are not removed (Supp. Methods in S1 Text). All methods use a permissive overlap definition [61] (Supp. Methods in S1 Text), except for the Standard Pfam (there overlaps of even one amino acid are removed [14]). The Standard Pfam thresholds are mapped to *p*-values, *q*-values, and lFDRs, and the medians of these distributions are used in comparisons (Supp. Results and Fig N in S1 Text).

### Empirical FDR tests

We introduce a suite of tests that measure empirical FDRs using biologically-motivated definitions of TPs and FPs. The “standard” biological sequence null model, which most software from BLAST to HMMER use, consists of random sequences generated assuming independent and identically distributed amino acids. Domains predicted on these random sequences produce a distribution of random bit scores from which *p*-values are computed. The five empirical tests we use instead label every prediction as either a TP or a FP, and these labels are used to compute empirical FDRs and *E*-values (number of type I errors, or FPs). Each test makes different assumptions, and together they provide independent and complementary evaluations. We describe our two primary tests in detail next; for the other three, see Supp. Methods (S1 Text).

#### Clan Overlap (ClanOv)

This test is inspired by [46]. After domains are predicted on a sequence and ranked by *p*-value, only overlaps between domains of the same clan are eliminated. Each remaining domain is labeled a FP if it overlaps a higher-ranking domain of a different clan, and otherwise it is labeled a TP. The “permissive” overlap definition is used (Supp. Methods in S1 Text). Overlaps are removed before counting domains (*y*-axis of plots such as Fig 3). Since *q*-values and lFDRs are computed on domains without overlaps, but ClanOv requires overlaps to measure empirical FDRs, here domains that overlap higher-ranking domains must be preserved and must have *q*-values and lFDRs, which are assigned by interpolation. This test does not evaluate the Standard Pfam fairly, which gets an FDR of zero, partly because the Standard Pfam thresholds are directly optimized on a similar test to prevent inter-clan overlaps [14], but also because our “nesting” list of allowed overlaps is defined using the Standard Pfam (Supp. Methods in S1 Text).

#### Context Coherence (ContextC)

This test extends one that previously used domain co-occurrence to estimate the FDR [47]. Here, given a list *L* of context family pairs (families that co-occur within the same sequence) and domains ranked by *p*-value, a domain is labeled as a TP if it is the highest-ranking domain or a higher-ranking domain can be found such that their family pair is in *L*; otherwise it is labeled a FP. This test does not evaluate the Standard Pfam fairly, which gets an FDR of zero, since *L* is defined by the Standard Pfam observations (Supp. Methods in S1 Text).

### Computing empirical FDRs

Given domain predictions labeled as either TPs or FPs as above, we compute empirical FDRs at two levels. Briefly, the “method-level” FDR evaluates an entire scoring method (*q*-values, *E*-values, etc.) combining all domain families, whereas the “family-level” FDR evaluates the accuracy of *q*-values separately per family. These quantities are consistent estimators of the corresponding true pFDRs under weak dependence [44]. At a threshold *t*, let TP_*ij*_(*t*) and FP_*ij*_(*t*) be the observed number of true positives and false positives, respectively, for domain family *j* in protein sequence *i*.

#### Method-level FDR

The empirical protein-level FDR, or epFDR, of a protein *i* with predictions, combines all domain families *j*,

$${\text{epFDR}}_{i}\left(t\right)=\frac{\sum_{j}{\text{FP}}_{ij}\left(t\right)}{\sum_{j}\left({\text{TP}}_{ij}\left(t\right)+{\text{FP}}_{ij}\left(t\right)\right)}.$$

The method-level empirical FDR is the mean epFDR_i_(*t*) over all proteins *i* with predictions, which corresponds to the expected FDR per protein. This per-protein FDR normalizes for domain counts, so proteins with hundreds of domain instances are weighted the same as proteins with fewer domains. Similarly, the empirical *E*-value is $\sum_{i}\sum_{j}{\text{FP}}_{ij}\left(t\right)$ across all proteins and families. The standard errors used in plots are computed from the epFDR_i_(*t*) and $\sum_{j}{\text{FP}}_{ij}\left(t\right)$ distributions over proteins, for FDRs and *E*-values respectively.

#### Family-level FDR

This procedure measures per-family deviations between the empirical FDRs and the *q*-value threshold of 1e-2; ideally they agree. The empirical family-level FDR, or efFDR, of family *j* is defined as

$${\text{efFDR}}_{j}\left(q\right)=\frac{1+\sum_{i}{\text{FP}}_{ij}\left(q\right)}{1+\sum_{i}\left({\text{TP}}_{ij}\left(q\right)+{\text{FP}}_{ij}\left(q\right)\right)}\;,$$

combining observations across proteins *i*, and the log-deviation is defined as

$${\text{LD}}_{j}={\text{log}}_{2}\frac{{\text{efFDR}}_{j}\left(q\right)}{q}.$$

A pseudocount of 1 is used in efFDR_j_(*q*) so LD_j_ is defined when there are no observed FPs. For families with few predictions, the LD may be artificially large or small. We compute a two-tailed *p*-value (*p*_*Poisson*_) of the empirical *E*-value $\sum_{i}{\text{FP}}_{ij}\left(q\right)$ using the Poisson distribution with parameter $q\sum_{i}\left({\text{TP}}_{ij}\left(q\right)+{\text{FP}}_{ij}\left(q\right)\right)$, which is the expected number of FPs given the number of observations, excluding the pseudocount. The *p*_*Poisson*_ distribution across families is used to compute *q*-values (*q*_*Poisson*_, unrelated to the domain *q*-value threshold), and a measurement is deemed significant if *q*_*Poisson*_ *≤* 1e-3. Families are also separated by effect size: positive deviations if LD_j_ > 2, negative deviations if LD_j_ < -2, and small deviations if |LD_j_| *≤* 2.

### Domain Prediction Using Context (dPUC)

DPUC improves domain prediction by taking into account the “context,” or presence of other domain predictions [48]. A newer version of dPUC now works with HMMER3, among other improvements that will be described elsewhere. Context family pair counts were derived from Pfam 25 on UniProt proteins. The “candidate domain *p*-value threshold” of dPUC is a tunable parameter, which when set to *p ≤* 1e-4 gives comparable empirical FDRs to *q ≤* 4e-4 on the MarkovR and OrthoC tests. dPUC is not evaluated in ContextC because both are based on domain context (dPUC would have a zero empirical FDR), nor in ClanOv because dPUC requires overlap removal while ClanOv requires observing overlaps to compute its FDR. DPUC 2.0 is available at http://compbio.cs.princeton.edu/dpuc.

### Categorizing families with repetitive patterns

PairCoil2 [62], TMHMM [63], and SEG [64], were run on UniRef50 using standard parameters to predict coiled coils, transmembrane domains, and low-complexity regions, respectively. Each Pfam family observed at least 4 times in UniRef50 was associated with a category if more than half of its domains overlapped the category's predictions. For families with multiple categories, only the one with the greatest amino acid overlap was kept. Unassigned families were categorized as “other”.

## Supporting Information

S1 Text Supplementary information. Contains the supplementary methods, results, tables and figures.
(PDF)

S1 File Pfam families with as-expected noise. Table with family accessions and annotation, number of “votes” (how many tests declare it as having an insignificant or small effect size difference from the expected FDR), HMM length, and “type” is the repetitive pattern category: T = transmembrane domain, L = low-complexity region, C = coiled coil, and N = other (normal).
(TXT)

S2 File Pfam families with increased noise. Table with family accessions and annotation, number of “votes” (how many tests declare it as having a significantly large positive effect size difference from the expected FDR), HMM length, and “type” is the repetitive pattern category: T = transmembrane domain, L = low-complexity region, C = coiled coil, and N = other (normal).
(TXT)

S3 Files Random protein sequences, based on a 2nd order Markov model derived from UniRef50. This dataset is used for one of our empirical FDR tests (see S1 Text).
(FA) Click here for additional data file.

S4 Files Random protein sequences, based on a 2nd order Markov model derived from UniRef50. This dataset is used for one of our empirical FDR tests (see S1 Text).
(FA) Click here for additional data file.

S5 Files Random protein sequences, based on a 2nd order Markov model derived from UniRef50. This dataset is used for one of our empirical FDR tests (see S1 Text).
(FA) Click here for additional data file.

S6 Files Random protein sequences, based on a 2nd order Markov model derived from UniRef50. This dataset is used for one of our empirical FDR tests (see S1 Text).
(FA) Click here for additional data file.

S7 Files Random protein sequences, based on a 2nd order Markov model derived from UniRef50. This dataset is used for one of our empirical FDR tests (see S1 Text).
(FA) Click here for additional data file.

S8 Files Random protein sequences, based on a 2nd order Markov model derived from UniRef50. This dataset is used for one of our empirical FDR tests (see S1 Text).
(FA) Click here for additional data file.

S9 Files Random protein sequences, based on a 2nd order Markov model derived from UniRef50. This dataset is used for one of our empirical FDR tests (see S1 Text).
(FA) Click here for additional data file.

S10 Files Random protein sequences, based on a 2nd order Markov model derived from UniRef50. This dataset is used for one of our empirical FDR tests (see S1 Text).
(FA) Click here for additional data file.
