# Supplementary material for: Beyond the E-Value: Stratified Statistics for Protein Domain Prediction
Source: PLoS Comput Biol. 2015 Nov 17;11(11):e1004509. doi: 10.1371/journal.pcbi.1004509 (PMC4648515; doi:10.1371/journal.pcbi.1004509)
Supplement: S1 Text — Contains the supplementary methods, results, tables and figures. (PDF) [file pcbi.1004509.s001.pdf]

Supplementary methods

Construction of *q*-value estimates. Given *p*-values and a threshold *t*, the pFDR is estimated as

$$\widehat{\mathrm{pFDR}}(t) = \frac{t\hat{\pi}_0 N}{n(t)} ,$$

where $\hat{\pi}_0$ is the estimated proportion of tests where the null hypothesis holds, *N* is the number of tests, and *n*(*t*) is the number of tests declared significant [1–3]. This estimate assumes correct *p*-values, which are uniformly distributed when the null hypothesis holds. The estimated *q*-value of *p* is then

$$\hat{q}(p) = \min_{t \geq p} \widehat{\mathrm{pFDR}}(t) ,$$

which assigns the lowest pFDR estimate that will reject the null hypothesis for this *p*-value.

Construction of lFDR estimates. The local FDR (lFDR) corresponds to the posterior probability that a prediction with a given *p*-value is false [3,4]. Our lFDR estimate is

$$\widehat{\mathrm{lFDR}}(t) = \frac{\hat{\pi}_0}{\hat{f}(t)} ,$$

where $\hat{f}(t)$ is an estimate of the *p*-value density and $\hat{\pi}_0$ is as for *q*-values. The density is difficult to estimate because the observed *p*-values are discrete observations. We use the Grenander density estimator for $\hat{f}(t)$ [3], which is the non-parametric maximum likelihood estimator of the density assuming that it decreases monotonically [5]. We implemented the pool-adjacent-violators (PAV) algorithm [6] to find the Grenander density. Additionally, prior to the Grenander density estimation, the empirical cumulative *n*(*t*) (same as for *q*-values) must be adjusted to satisfy

$$0 \leq \frac{n(t)}{N} - \hat{\pi}_0 t \leq 1 - \hat{\pi}_0 , \quad (1)$$

to ensure consistency between $\hat{\pi}_0$ and the two-component model [3].

Removal of domain overlaps for better FDR estimates. Our *q*-values and lFDRs are only correct if they are computed after removing domain overlaps, otherwise noise is underestimated because the number of predictions is overestimated. We remove all overlaps between domains by only keeping those with the smallest *p*-values; while this worked well in practice, future research should address whether more powerful approaches to overlap removal exist.

Adaptation of *q*-value and lFDR methods for filtered *p*-values. Existing implementations of *q*-value (R package qvalue) and lFDR (R packages qvalue and fdrtool) require a complete list of *p*-values (to estimate π_0), which HMMER3 does not provide [7]. We reimplemented the algorithms to overcome this problem, defining the necessary parameters in other ways while leaving most of the procedures unchanged. The number of tests *N* is the number of proteins for sequence *p*-values, and for domain *p*-values it is the number of proteins plus the additional number of domains predicted in each sequence when there are more than one. The implied value $n(1)=N$ is added to ensure that $\hat{q}(t) \leq 1$ and $\widehat{\mathrm{lFDR}}(t) \leq 1$. The π_0 cannot be estimated because the distribution of *p*-values near 1 is necessary [1–3]. We set $\hat{\pi}_0 = 1$, a reasonable approximation since any given family is not present in the majority of proteins. Lastly, the constraints of eq. 1 are replaced with $N t \leq n(t)$. Beside these changes, *q*-values and lFDRs are estimated as described before.

Equal stratified lFDRs are also optimal when controlling the combined *E*-value. We want to maximize the number of predictions, as in the **Results** (consult for notation), but here we constrain the combined *E*-value to a maximum value of *E*, or

$$\sum_i t_i \pi_{0,i} N_i \leq E ,$$

and the Lagrangian multiplier function is then

$$\Lambda = \sum_i F_i(t_i) N_i + \lambda \left(\sum_i t_i \pi_{0,i} N_i - E \right),$$

where only Λ and λ differ from those of the FDR problem. A necessary condition for optimality is

$$\frac{\partial \Lambda}{\partial t_j} = f_j(t_j) N_j + \lambda \pi_{0,j} N_j = 0 \;\Leftrightarrow\; -\frac{1}{\lambda} = \frac{\pi_{0,j}}{f_j(t_j)} = \mathrm{lFDR}_j(t_j),$$

again showing that the lFDR of each stratum must be equal.

Method rankings are preserved between *E*-value and FDR plots. We present the outline of the proof that method rankings are preserved in plots with the number of predictions on the *y*-axis and either the FDR or *E*-value on the *x*-axis.

a) Definition. Given two methods *A*, *B* with functions f_A, f_B in the same domain *X*, *A* ranks higher than *B* if $f_A(x) \geq f_B(x)$ for all *x* in *X*.

b) Theorem. Method *A* ranks higher than *B* with respect to FDRs if and only if *A* ranks higher than *B* with respect to *E*-values.

c) Proof. First, observe that if both f_A and f_B are invertible and monotonically increasing, *A* ranks higher than *B* implies that the inverse of *A* ranks lower than the inverse of *B*. The FDR and *E*-value functions are monotonically increasing, and for simplicity we assume them to be invertible (although real data is discrete, for a given number of predictions, an inverse function can be defined that returns the lowest FDR or *E*-value when there are ties, and we obtain a continuous function by interpolation). Thus, it suffices to show that rankings are preserved in the inverse functions when we go from FDRs to *E*-values.

For a given method *A*, its inverse FDR and *E*-value curves $\mathrm{FDR}_A(n)$ and $E_A(n)$, which are functions of the number of predictions *n*, are related by

$$\mathrm{FDR}_A(n) = E_A(n)/n.$$

It follows that the ranking of the inverses of the two methods *A* and *B* is preserved, because

$$\mathrm{FDR}_A(n) \geq \mathrm{FDR}_B(n) \;\Leftrightarrow\; E_A(n) \geq E_B(n),$$

which follows since $n>0$.

Tiered stratified *q*-value thresholds. Under a simple independence assumption, the final FDR of tiered *q*-values is approximately the sum of the per-tier *q*-value thresholds if both are small.

The final $\mathrm{FDR}_{\mathrm{tiered}}$ is characterized in terms of Bayesian posterior error rates [8], and the joint distribution of sequence *q*-values (q_{seq}) and domain *q*-values conditional on the sequence threshold ($q_{\mathrm{dom|seq}}$). A significant domain prediction satisfies both thresholds Q_{dom} and $Q_{\mathrm{dom|seq}}$, it is truly false if the null hypothesis is true for either the sequence-level ($H_{\mathrm{seq}}=0$) or domain-level ($H_{\mathrm{dom}}=0$) tests, and in this model the probability of these events is the same for every significant prediction [8]. Therefore,

$$\begin{aligned}
\mathrm{FDR}_{\mathrm{tiered}} &= \Pr(H_{\mathrm{seq}}=0 \vee H_{\mathrm{dom}}=0 \mid q_{\mathrm{seq}} \leq Q_{\mathrm{seq}} \wedge q_{\mathrm{dom|seq}} \leq Q_{\mathrm{dom|seq}}) \\
&= 1 - \Pr(H_{\mathrm{seq}}=1 \wedge H_{\mathrm{dom}}=1 \mid q_{\mathrm{seq}} \leq Q_{\mathrm{seq}} \wedge q_{\mathrm{dom|seq}} \leq Q_{\mathrm{dom|seq}}) \\
&= 1 - \Pr(H_{\mathrm{seq}}=1 \mid q_{\mathrm{seq}} \leq Q_{\mathrm{seq}} \wedge q_{\mathrm{dom|seq}} \leq Q_{\mathrm{dom|seq}}) \Pr(H_{\mathrm{dom}}=1 \mid q_{\mathrm{seq}} \leq Q_{\mathrm{seq}} \wedge q_{\mathrm{dom|seq}} \leq Q_{\mathrm{dom|seq}}) \qquad (2) \\
&= 1 - \Pr(H_{\mathrm{seq}}=1 \mid q_{\mathrm{seq}} \leq Q_{\mathrm{seq}}) \Pr(H_{\mathrm{dom}}=1 \mid q_{\mathrm{seq}} \leq Q_{\mathrm{seq}} \wedge q_{\mathrm{dom|seq}} \leq Q_{\mathrm{dom|seq}}) \qquad (3) \\
&= 1 - (1 - \Pr(H_{\mathrm{seq}}=0 \mid q_{\mathrm{seq}} \leq Q_{\mathrm{seq}}))(1 - \Pr(H_{\mathrm{dom}}=0 \mid q_{\mathrm{seq}} \leq Q_{\mathrm{seq}} \wedge q_{\mathrm{dom|seq}} \leq Q_{\mathrm{dom|seq}})) \\
&= 1 - (1 - Q_{\mathrm{seq}})(1 - Q_{\mathrm{dom|seq}}) \\
&= Q_{\mathrm{seq}} + Q_{\mathrm{dom|seq}} - Q_{\mathrm{seq}} Q_{\mathrm{dom|seq}}.
\end{aligned}$$

Above we assume that H_{dom} and H_{seq} are conditionally independent (eq. 2), and we use the fact that H_{seq} is independent of the domain threshold (eq. 3). Thus, $\mathrm{FDR}_{\mathrm{tiered}} \approx Q_{\mathrm{seq}} + Q_{\mathrm{dom|seq}}$ for small per-tier thresholds, since $Q_{\mathrm{seq}} Q_{\mathrm{dom|seq}}$ is negligible. The full equation is an approximation that breaks down in extreme cases when H_{dom} and H_{seq} are not conditionally independent (for example, $Q_{\mathrm{seq}}=1$ should simplify to single-tier domain thresholds and we should have $\mathrm{FDR}_{\mathrm{tiered}}=Q_{\mathrm{dom|seq}}$, but these equations instead predict $\mathrm{FDR}_{\mathrm{tiered}}=1$; also, $Q_{\mathrm{seq}}=0$ and $Q_{\mathrm{dom|seq}}=0$ separately give $\mathrm{FDR}_{\mathrm{tiered}}=Q_{\mathrm{dom|seq}}$ and $\mathrm{FDR}_{\mathrm{tiered}}=Q_{\mathrm{seq}}$ respectively, but $\mathrm{FDR}_{\mathrm{tiered}}$ should go to zero for both cases, in the limit that fewer predictions are made).

However, our assumption of conditional independence is reasonable for less extreme cases, and it performs well empirically.

Sequence datasets. Here UniProt [9] is version 2010_05 (11,384,036 proteins), which accompanies Pfam 25. UniRef50 is version 2011_04 (11 months newer than UniProt above; 3,865,311 proteins), and is obtained by filtering UniProt 2011_04 to 50% identity with CD-HIT [10]. OrthoMCL5 is the set of proteins of OrthoMCL [11] version 5 with pseudogenes removed, then each orthologous group was filtered to 50% identity with CD-HIT, removing proteins without domains matching across any ortholog with domain $p \leq 1\text{e-}4$ (see OrthoC in **Methods**) and singleton groups, leading to 379,010 proteins in 58,129 final orthologous groups.

Pfam. We use the 12,273 HMMs and curated thresholds ("Standard Pfam") of Pfam 25. However, we extend and update the Pfam clan definitions with those of Pfam 26 to improve the quality of our empirical FDR tests (particularly ClanOv). Since the Standard Pfam thresholds correspond to a fixed FDR, they are "extended" by shifting them by constant amounts as described previously [12], allowing us to explore a range of FDRs.

Permissive overlaps. To avoid domain loss due to errors in domain boundaries, we define overlaps "permissively": two domains overlap if the intersection of their ranges is larger than 40 amino acids or larger than 50% of the smallest of the two ranges [13].

Nested families. Two families in the "nesting" network are connected if their domains may overlap without removal of either, and both may be TPs in the ClanOv test. We allow all overlaps between these families, not just strict nesting. Our nested families are those that overlap with the Standard Pfam thresholds on UniProt and UniRef50, extended using clans: if A-B is in the network, and C and D are of the same clan as A and B, respectively, then C-D is added to the network.

Context families. Family pairs observed with the Standard Pfam thresholds without overlaps on UniProt and UniRef50 form our context network *L*, extended using clans: if A-B is in *L*, and C and D are in the same clan as A and B, respectively, then C-D is added to *L*.

Domain information content score based on the Gene Ontology. We reimplemented the MultiPfam2GO [14] procedure in-house to be more efficient. In summary, we computed statistical associations between Gene Ontology (GO) [15] terms version 2011-09-20 on UniProt and domain sets from Pfam 25 on UniProt. GO terms from all three standard ontologies were used. A naive Bayes classifier for each GO term uses a protein's domain content to predict whether the sequence is annotated by the term. The information content [16] of each GO term *i* is

$$I_i^{GO} = -\log_2 p_i \,,$$

where p_i is the fraction of proteins with GO terms in UniProt that have GO term *i*. The "GO information content" of a domain architecture is the sum of I_i^{GO} of leaf GO terms only.

Ortholog Set Coherence (OrthoC). This test extends our previously-published "Ortholog Coherence" test [12]. Given a set of orthologs from OrthoMCL5 [11], a domain predicted in one sequence (after setting thresholds and removing overlaps) is a TP if any domain of the same clan is present anywhere in at least one other ortholog with $p \leq 1\text{e-}4$; it is a FP otherwise (**Fig A**). To prevent high-identity sequence pairs from producing correlated FPs that supports each other and are scored as TPs, we used CD-HIT on each group of orthologs so that no two sequences had greater than 50% identity [10]. Lastly, proteins with every domain labeled FP at $p \leq 1\text{e-}4$ are removed from the test (we assume they are false orthologs).

Second-order Markov random sequences (MarkovR). This test extends the standard random sequence null model, and is an improved version of our previously published test [12]. In summary, random sequences are constructed using a second-order Markov model derived from UniRef50, which generate null domains (**Fig A**). This random sequence model preserves local amino acid correlations and should better model low-complexity regions than the standard random sequence null model, which assumes independence between positions.

The mth order Markov model is parametrized by the equivalent *k*-mer distribution with $k=m+1$, which is obtained from sequences with these filters: we ignore the initial M (methionine) when present, ignore *k*-mers with ambiguous amino acid codes (B,J,X,Z), and replace the rare amino acids U and O with C and K, respectively. Random sequences are initialized with M and a random *k*-mer, and every following amino acid is chosen from the conditional distribution given the previous (*k*-1)-mer. The sequence terminates when the desired length is reached. Our RandProt 1.0 software, which generates these random protein sequences, is available at https://github.com/alexviiia/RandProt.

Every real sequence is matched to a random sequence of the same length, and their domain predictions are combined with their ranges preserved. Domains from the real sequence are TPs, and from the random sequence are FPs. Each method produces predictions on this set by setting thresholds and removing overlaps.

We assume FPs arise in real sequences with equal frequency as in random sequences. However, every domain from a real sequence will be counted as a TP; we double the measured FDR (and *E*-value) to correct for this effect. More formally, we measure quantities TP', FP', which are related to the true quantities by E[FP'] = FP/2 and E[TP'] = TP+FP/2, the second of which follows since the total prediction counts are fixed, that is TP'+FP'=TP+FP. Applying these transformations to the pFDR, treating the denominator as fixed, gives E[pFDR']=pFDR/2, and thus doubling is necessary.

Reverse Sequence (RevSeq). This test is based on the fact that the reverse of a protein sequence is very unlikely itself to be a functional protein, so it is a simple way of generating proteins without any true domains. Moreover, reversed sequences preserve repetitive and low-complexity regions, which are known to be problematic for homology prediction, so they may be used to generate more realistic null statistics than the standard null model (which generates repetitive and low-complexity regions more rarely) [17].

RevSeq has one problematic case: some domain families have truly symmetric subregions, which we do not wish to penalize. We address this problem by removing domain predictions from the reverse sequence that overlap (in the strict sense) predictions of the same family from the real sequence. Overlaps were determined by reversing the coordinates of the prediction from the reverse sequence, so that there is a subsequence that is predicting the same domain family in both the forward and reverse orientations. This filter removed 6.2% of the reverse sequence domain predictions. This FDR test proceeds identically to MarkovR, merging the domains from the real sequence with those from the reverse sequence (**Fig A**). The measured FDR is also adjusted by a factor of 2.

Supplementary results

The Standard Pfam thresholds in terms of stratified *q*-values and lFDRs. We want *q*-values and lFDR estimates that are comparable to the Standard Pfam bitscore thresholds. For each family, its *p*-values on UniRef50 are assigned *q*-values and lFDRs. The *p*-value of each family's bitscore threshold is given by the Extreme Value Distribution with parameters from each HMM file. Lastly, the bitscore is assigned a *q*-value and lFDR by linear interpolation.

It has been reported [18] that Pfam families with thresholds of 25 and 27 bits are the default threshold values (“uncurated”), and the rest of the families (“curated”) tend to have higher *p*-value thresholds. We observed a sharp *p*-value distribution for these thresholds, which is bimodal for the full data, and unimodal for the “curated” subset (median p=1.3e-8), in agreement with previous results [18] (**Fig N**). The *q*-value distribution of these thresholds is less sharp, and changes less when excluding the “uncurated” subset, with a “curated” median q=4.2e-4.

The lFDR distribution has modes at 0 and 1 (**Fig N**). 16% of Pfam families have $\mathrm{lFDR} \geq 0.5$, so their worst predictions should probably be false. However, the Pfam thresholds of these families fell on a region of the *p*-value distribution where there were few domains identified due to overlap removal (data not shown). Thus, our density estimates for these thresholds were flawed. We believe these

Standard Pfam thresholds have reasonable lFDRs but we are unable to compute them accurately. Threshold setting on observed data works well, since density estimation on the observed *p*-values is more accurate; only interpolation is a problem for thresholds of families with few or no smaller *p*-values observed. When these undesirable thresholds are excluded, we find a median "curated" lFDR threshold of 2.3e-2.

Pfam threshold curation somewhat compensates for underestimated noise. Here we investigate to what extent the Standard Pfam curated thresholds compensate for underestimated FDRs. We compare the *q*-value distributions of the Standard Pfam thresholds for the increased-noise and as-expected noise families separately. We find that increased-noise families have more stringent thresholds than as-expected-noise families under the Standard Pfam, with median *q*-values differing by a factor of 19. However, there is no clear separation between both *q*-value distributions (**Fig J**). In particular, 15% of the increased-noise families have a *q*-value threshold larger than the median as-expected-noise threshold. Overall, while Pfam has more stringent thresholds for increased-noise families than for families with as-expected noise, many increased-noise family thresholds remain more permissive than they should be.

Evaluation of empirical FDR tests. ClanOv identified the most increased-noise families, and empirical FDRs agree best with *q*-values in as-expected-noise families, as desired (**Fig K**). The 27 reduced noise families also had the largest differences between *q*-values computed before and after overlap removal (data not shown), so this subset may be an artifact: for *q*-values we remove all overlaps, but in ClanOv only within-clan overlaps are removed, leading to underestimated FDRs.

ContextC also identified increased-noise families, since observed domain pairs contain strong biological information. Increased-noise families are usually correctly assigned, since true domain pairs are almost always observed [19]. However, this test may underestimate the FDR, since it had the most decreased-noise families. One explanation is that true FP domains may often be singletons, which will be labeled erroneously as TPs. Therefore, this test is useful to study increased-noise, but not decreased-noise, families.

OrthoC successfully identified increased-noise families. However, its empirical FDRs had the least agreement with theory (**Fig 5**), even in families with as-expected noise (**Fig K**). Inspection suggests the problems are due to false ortholog assignments and orthologs with differing domain architectures, both of which mislabel TPs as FPs. Removal of the families with the most extreme deviations (|LD|>2) in OrthoC did not lead to better agreement between the empirical FDR and *q*-values (data not shown), so mislabeled domains did not concentrate in a few families. This test also has the smallest sample size (a third of UniRef50), since we only considered proteins with orthologs and with percent identities smaller than 50%. Therefore, OrthoC could be improved if ortholog predictions improve, if orthologs with differing architectures are identified and handled, and if the number of proteins increases.

RevSeq identifies many increased-noise families (**Fig 7**), and remarkably few decreased-noise families (**Table A**). The simplicity of these "random" sequences is also very appealing. However, we needed to address the case of true symmetry in domains (**Supp. Methods**) in order to get accurate empirical FDRs.

The MarkovR random sequences are very similar to those that theoretically generate the *p*-values: the latter is a 0th order Markov model. Empirical FDRs and *q*-values agreed very well (**Fig 5**). A 4th order Markov model behaves the same, but it risks including biological information (certain 5-mers are predictive of domain families; data not shown). Therefore, higher-order Markov models are unlikely to be useful in the future.

Supplementary references

1. Storey JD. A direct approach to false discovery rates. Journal of the Royal Statistical Society: Series B (Statistical Methodology). 2002;64: 479–498. doi:10.1111/1467-9868.00346
2. Storey JD, Tibshirani R. Statistical significance for genomewide studies. Proceedings of the National Academy of Sciences of the United States of America. 2003;100: 9440–9445. doi:10.1073/pnas.1530509100
3. Strimmer K. A unified approach to false discovery rate estimation. BMC Bioinformatics. 2008;9: 303. doi:10.1186/1471-2105-9-303
4. Efron B, Tibshirani R, Storey JD, Tusher V. Empirical Bayes Analysis of a Microarray Experiment. Journal of the American Statistical Association. 2001;96: 1151–1160. doi:10.1198/016214501753382129
5. Grenander U. On the theory of mortality measurement, Part II. Skan Aktuarietidskr. 1956;39: 125–153.
6. Barlow RE, Bartholomew DJ, Bremner JM, Brunk HD. Statistical inference under order restrictions; the theory and application of isotonic regression. New York: Wiley; 1972.
7. Eddy SR. HMMER3 is stubborn. In: Cryptogenomicon [Internet]. 19 Sep 2011 [cited 4 Jan 2013]. Available: http://selab.janelia.org/people/eddys/blog/?p=508
8. Storey JD. The positive false discovery rate: a Bayesian interpretation and the q-value. Ann Statist. 2003;31: 2013–2035. doi:10.1214/aos/1074290335
9. The UniProt Consortium. Reorganizing the protein space at the Universal Protein Resource (UniProt). Nucleic Acids Research. 2011; doi:10.1093/nar/gkr981
10. Li W, Godzik A. Cd-hit: a fast program for clustering and comparing large sets of protein or nucleotide sequences. Bioinformatics. 2006;22: 1658–1659. doi:10.1093/bioinformatics/btl158
11. Chen F, Mackey AJ, Jr CJS, Roos DS. OrthoMCL-DB: querying a comprehensive multi-species collection of ortholog groups. Nucleic Acids Res. 2006;34: D363–D368. doi:10.1093/nar/gkj123
12. Ochoa A, Llinás M, Singh M. Using context to improve protein domain identification. BMC Bioinformatics. 2011;12: 90. doi:10.1186/1471-2105-12-90
13. Yeats C, Redfern OC, Orengo C. A fast and automated solution for accurately resolving protein domain architectures. Bioinformatics. 2010;26: 745–751. doi:10.1093/bioinformatics/btq034
14. Forslund K, Sonnhammer ELL. Predicting protein function from domain content. Bioinformatics. 2008;24: 1681–1687. doi:10.1093/bioinformatics/btn312
15. Ashburner M, Ball CA, Blake JA, Botstein D, Butler H, Cherry JM, et al. Gene Ontology: tool for the unification of biology. Nat Genet. 2000;25: 25–29. doi:10.1038/75556
16. Resnik P. Using Information Content to Evaluate Semantic Similarity in a Taxonomy. Proceedings of the 14th International Joint Conference on Artificial Intelligence. 1995; Available: http://arxiv.org/abs/cmp-lg/9511007
17. Karplus K, Karchin R, Shackelford G, Hughey R. Calibrating E-values for hidden Markov models using reverse-sequence null models. Bioinformatics. 2005;21: 4107–4115. doi:10.1093/bioinformatics/bti629
18. Punta M, Coggill PC, Eberhardt RY, Mistry J, Tate J, Boursnell C, et al. The Pfam protein families database. Nucleic Acids Research. 2011;40: D290–D301. doi:10.1093/nar/gkr1065
19. Terrapon N, Gascuel O, Marechal E, Brehelin L. Fitting hidden Markov models of protein domains to a target species: application to Plasmodium falciparum. BMC Bioinformatics. 2012;13: 67. doi:10.1186/1471-2105-13-67

Supplementary tables

Table A. Size of domain family classes from per-family FDR analysis.

Test	Total families	Small deviation (|LD| ≤ 2)	%	Insignificant & |LD|>2	%	Significant & LD>2	%	Significant & LD<-2	%
ClanOv	12236	6606	53.99	4626	37.81	977	7.98	27	0.22
ContextC	12230	7138	58.36	4476	36.60	367	3.00	249	2.04
OrthoC	9051	2434	26.89	6161	68.07	456	5.04	0	0.00
RevSeq	12228	4619	37.77	6841	55.95	759	6.21	9	0.07
MarkovR	12230	6273	51.29	5945	48.61	0	0.00	12	0.10

Supplementary figures

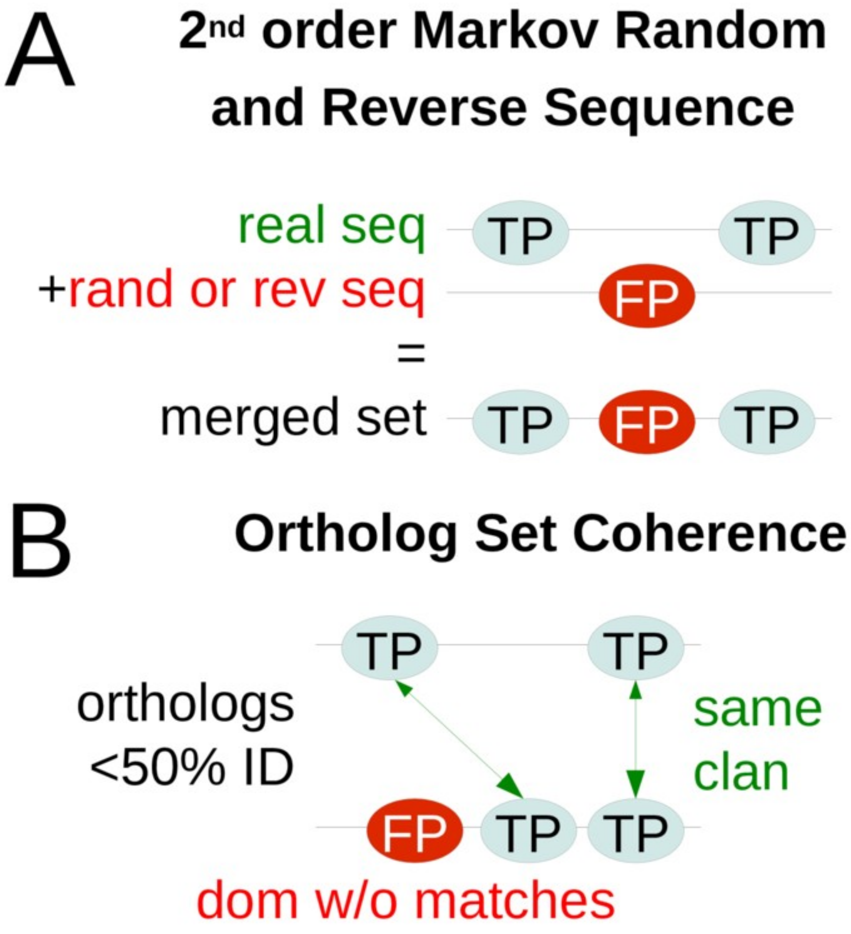

Figure A. Illustration of the empirical FDR tests MarkovR, RevSeq, and OrthoC. A. MarkovR (“Markov Random”) generates random sequences from a second-order Markov model, which is used to generate FP domains, while the domains from the real sequence are TPs. RevSeq (“Reverse Sequence”) is identical except the Markov sequence is replaced with the reverse sequence of the real protein. **B.** OrthoC (“Ortholog Set Coherence”) labels domains TPs if there are domains of the same clan in orthologs, otherwise they are FPs.

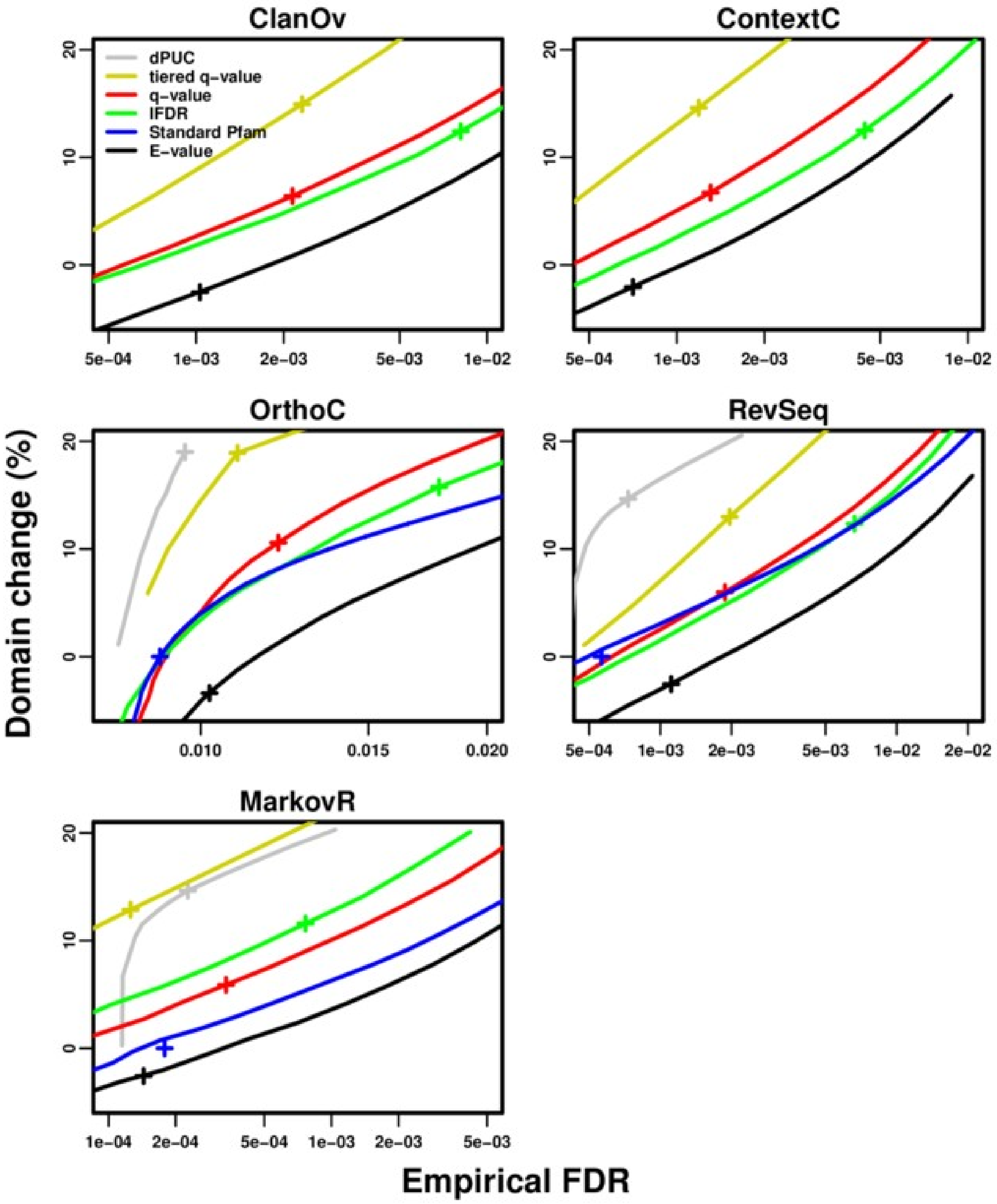

Figure B. Extended version of domain change while controlling for empirical FDRs. Extends **Fig 3** to include the OrthoC, RevSeq, and MarkovR tests, as well as the curves: the Standard Pfam (blue cross), extended to produce the blue curve (**Methods**); tiered *q*-value (yellow), cross marks the per-tier threshold of 1e-4; dPUC (gray), cross marks the "candidate domain *p*-value threshold" of 1e-4, which gives comparable empirical FDRs to the Standard Pfam (**Methods**). The blue cross is the only datapoint using strict overlap removal (of even a single amino acid), whereas the blue curve (and all other curves and crosses) use the permissive overlap definition described in the **Supp. Methods**; for that reason the blue cross tends to fall slightly under (instead of on) the blue curve.

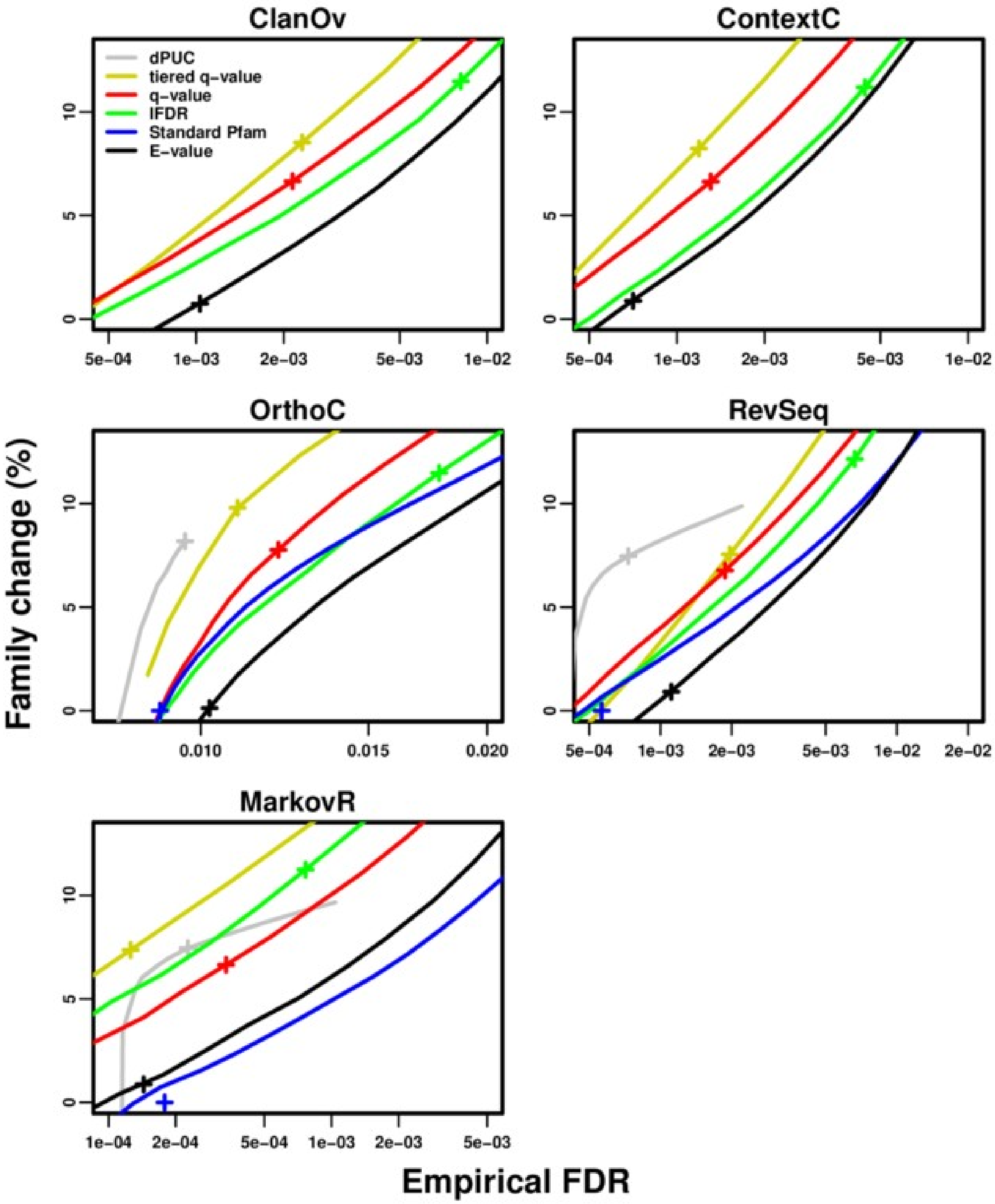

Figure C. Family change while controlling for empirical FDRs. Like **Fig B** except that the percent change of unique families per protein relative to the Standard Pfam is plotted on the *y*-axis. For example, for a protein with domain architecture A-B-B-B, **Fig B** counts this protein as having four domains, but this figure counts only two unique domain families, namely A and B. See **Fig 3** and **Fig B**.

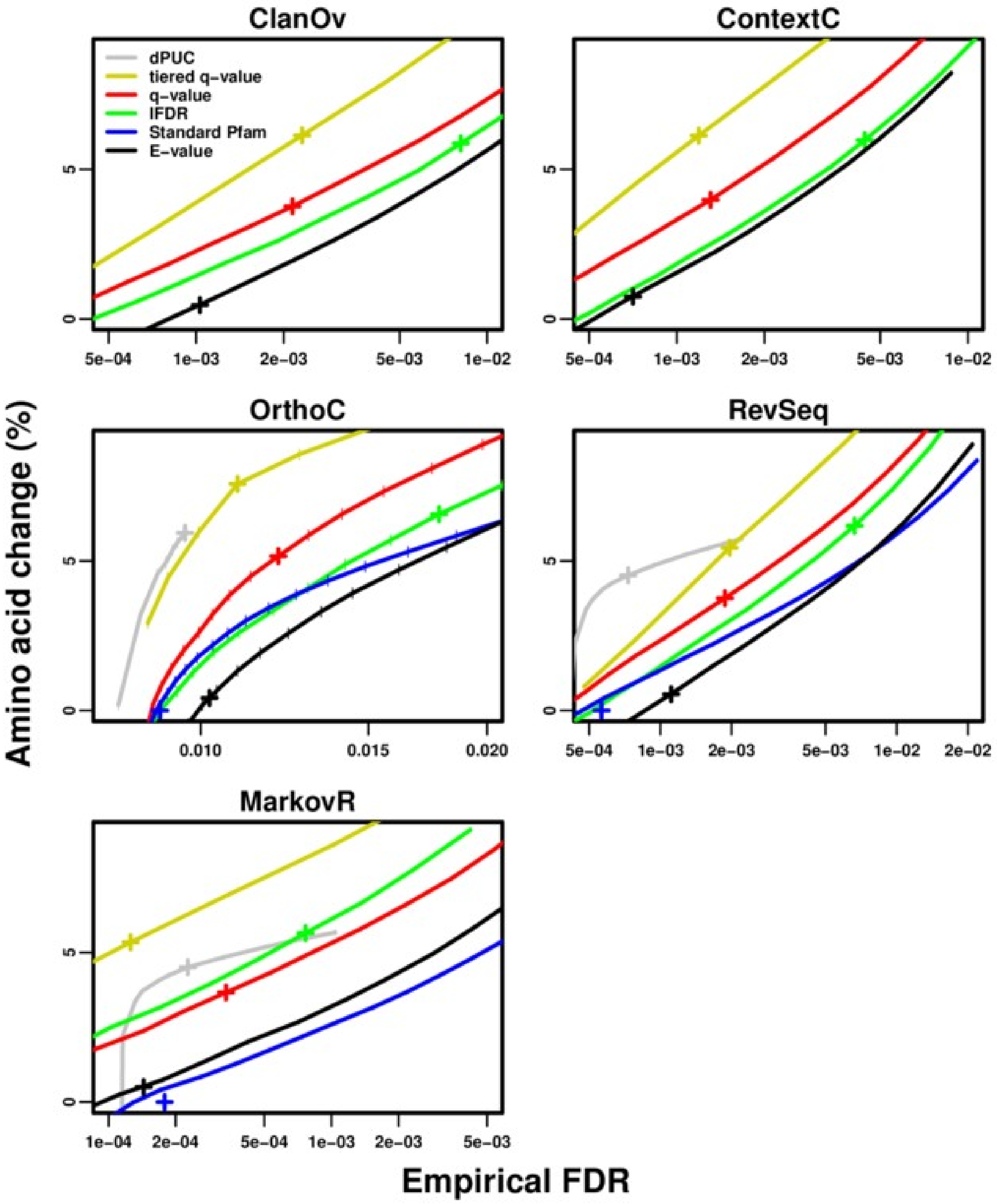

Figure D. Amino acid coverage change while controlling for empirical FDRs. Like **Fig B** except that the percent change of amino acid coverage across proteins relative to the Standard Pfam is plotted on the *y*-axis. See **Fig 3** and **Fig B**.

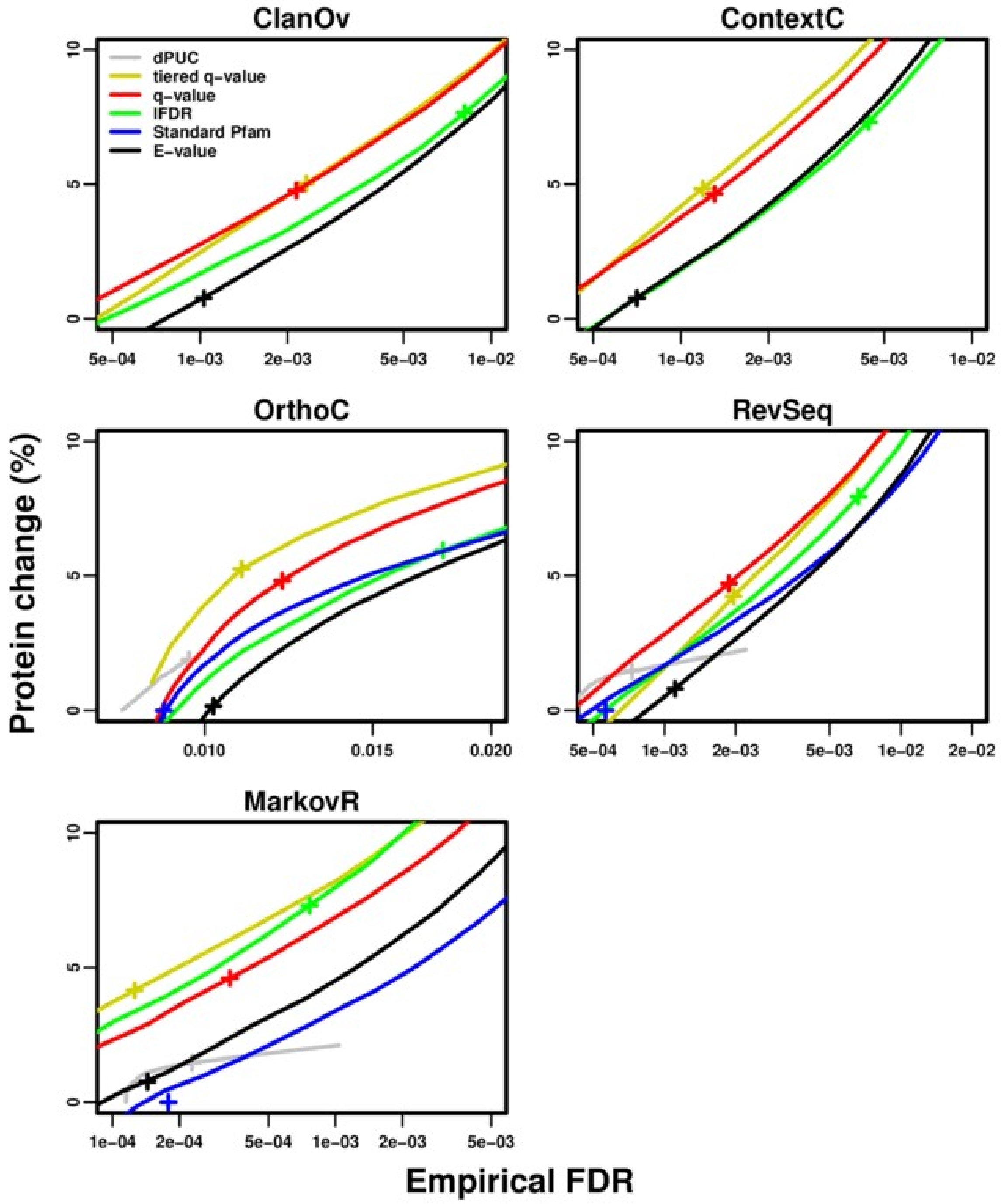

Figure E. Protein coverage change while controlling for empirical FDRs. Like **Fig B** except that the percent change of number of proteins with domains relative to the Standard Pfam is plotted on the *y*-axis. That is, here a protein is counted as an increase for each of these methods if it had at least one domain prediction under the given method but it did not have any domains under the Standard Pfam. Proteins can also be lost, and counted negatively, if a method did not predict any domains but the Standard Pfam did. See **Fig 3** and **Fig B**.

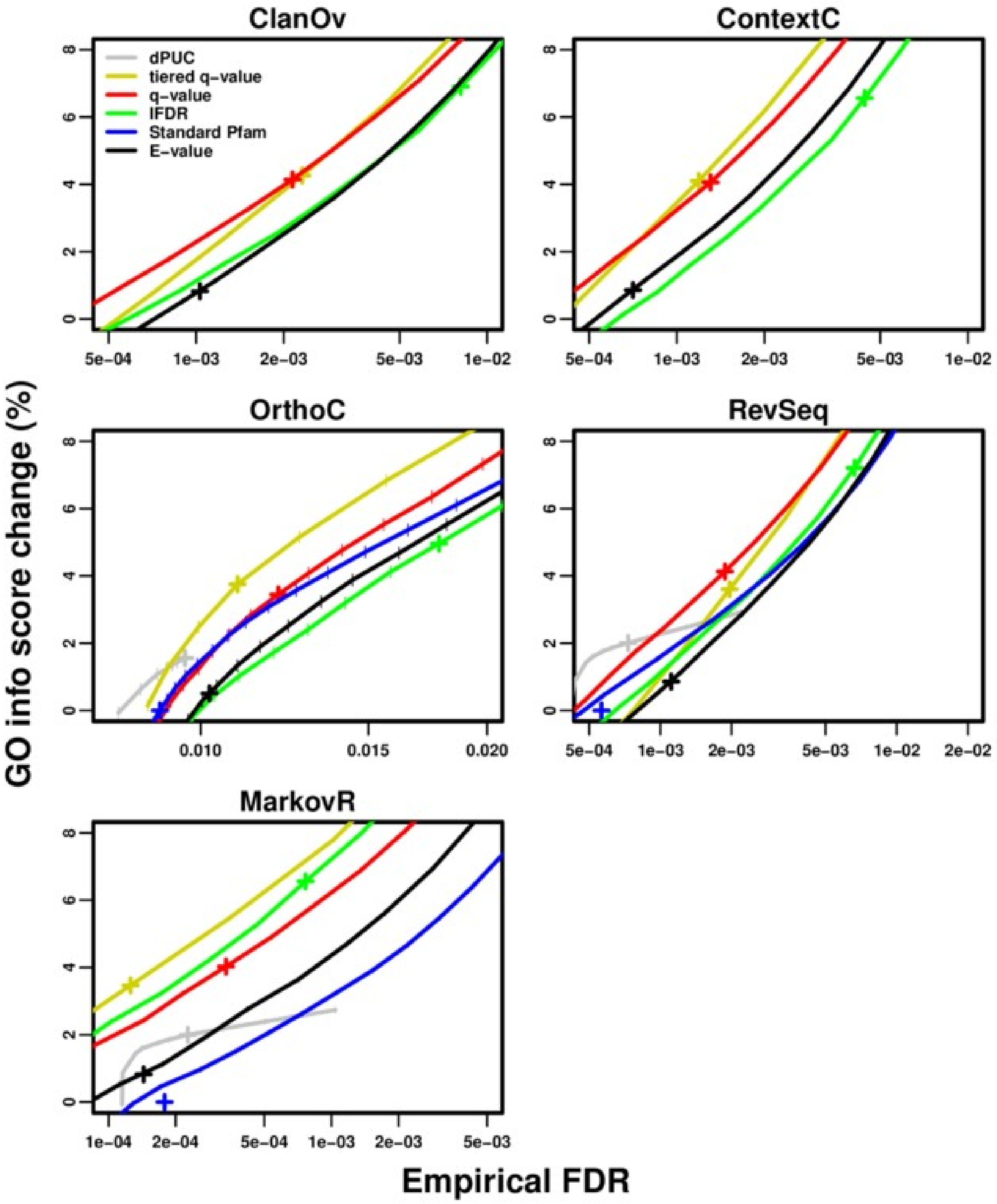

Figure F. GO information content change while controlling for empirical FDRs. Like **Fig B** except the change of the Gene Ontology information content (**Supp. Methods**) added across proteins relative to the Standard Pfam is plotted on the *y*-axis. See **Fig 3** and **Fig B**.

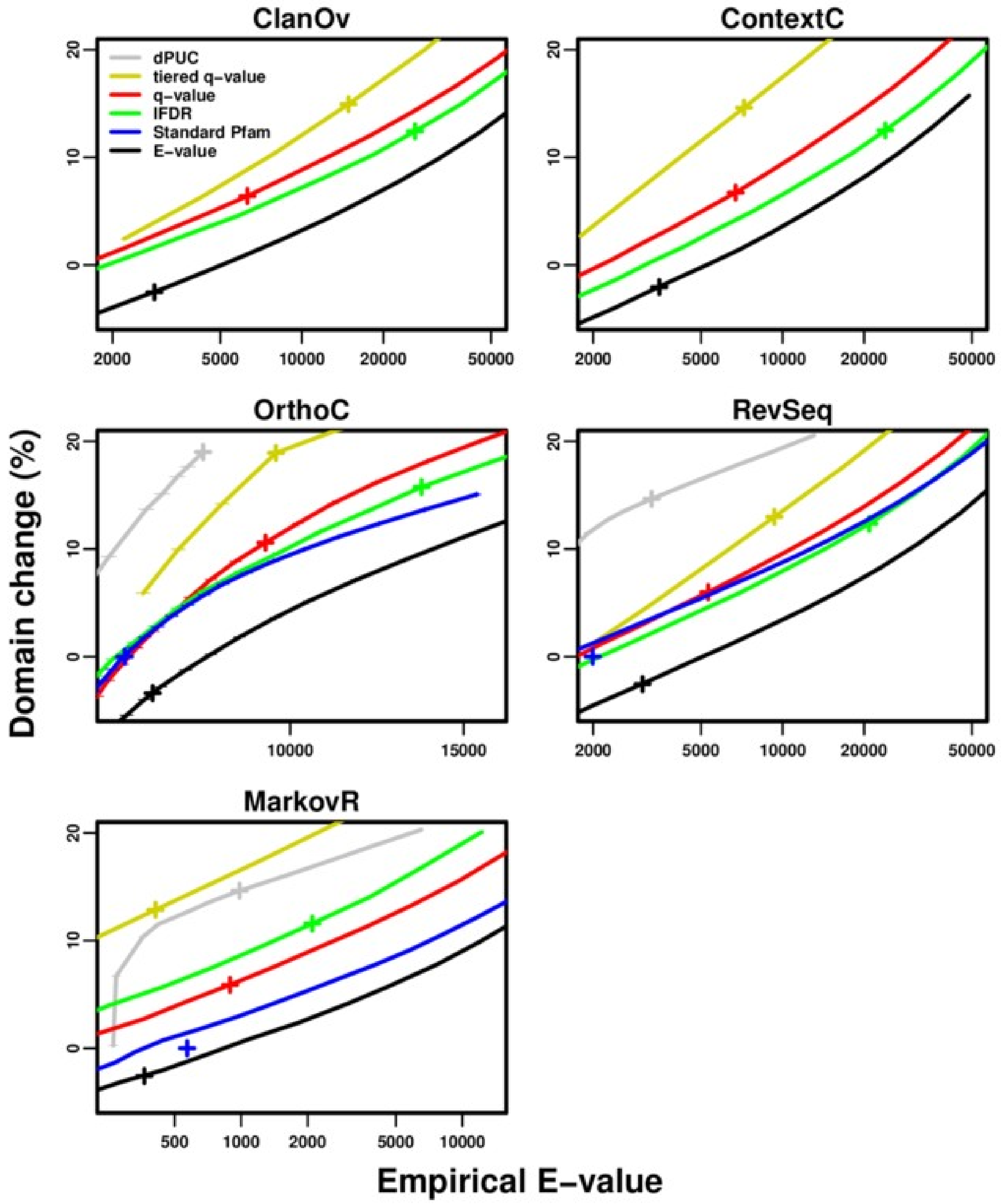

Figure G. Domain change across methods while controlling for empirical *E*-values. Like **Fig B** except that the *x*-axis plots empirical *E*-values instead of empirical FDRs. Unlike FDRs, which are always in [0,1], *E*-values are bounded by the number of *p*-values across domain families in each dataset, roughly equal to the number of domain families times the number of proteins. The number of families is the same across panels, but the number of proteins varies, with OrthoC having many fewer proteins than the other tests. See **Fig 3** and **Fig B**.

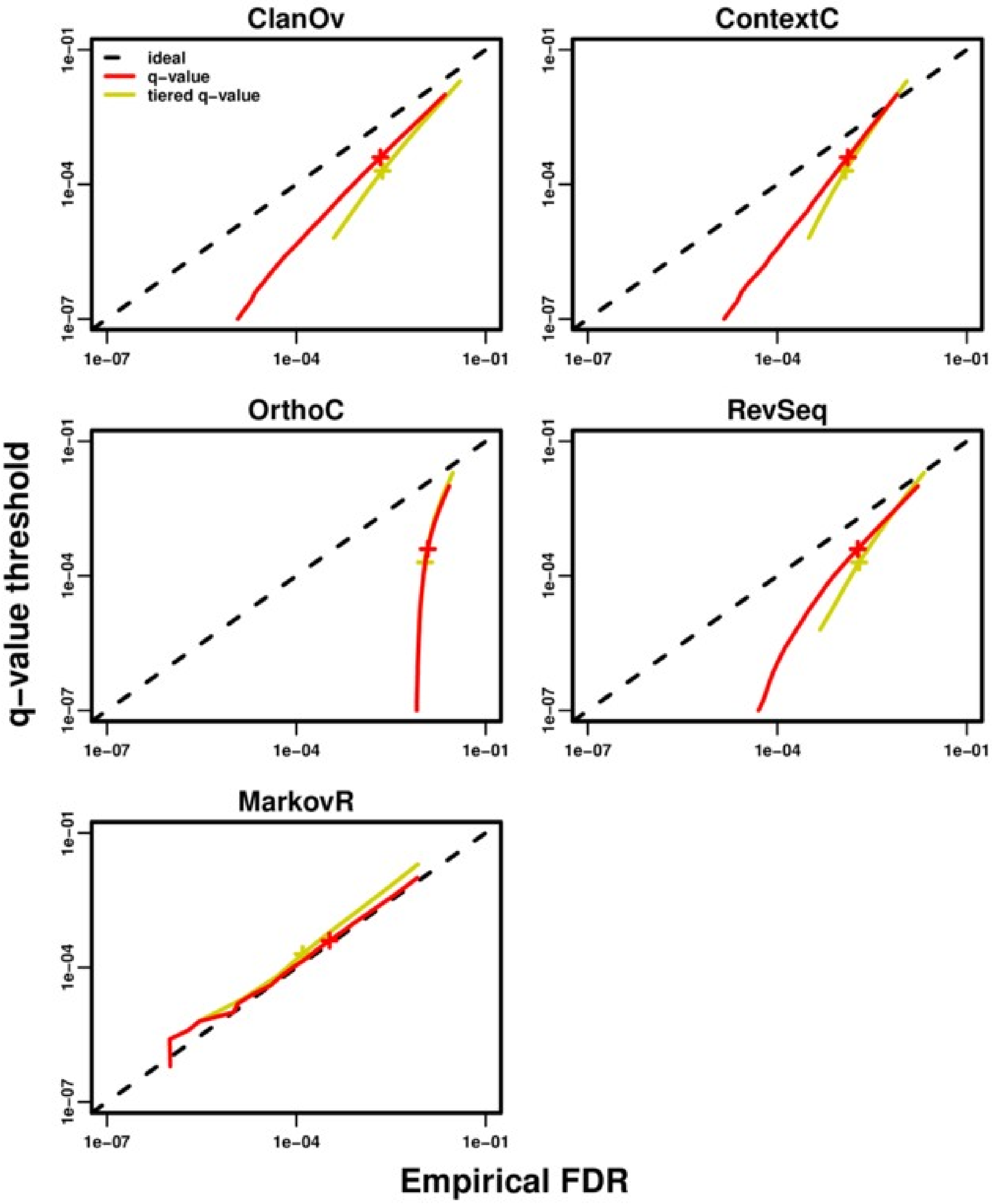

Figure H. Extended version of comparison of *q*-value thresholds and empirical FDRs. Extends **Fig 5** to include the OrthoC, RevSeq, and MarkovR tests. Also adds tiered stratified *q*-values (yellow, cross marks per-tier threshold of 1e-4), in which the theoretical FDR on the *y*-axis ("*q*-value threshold") corresponds to the per-tier threshold multiplied by 2 in accordance with a theoretical result from the **Supp. Methods**.

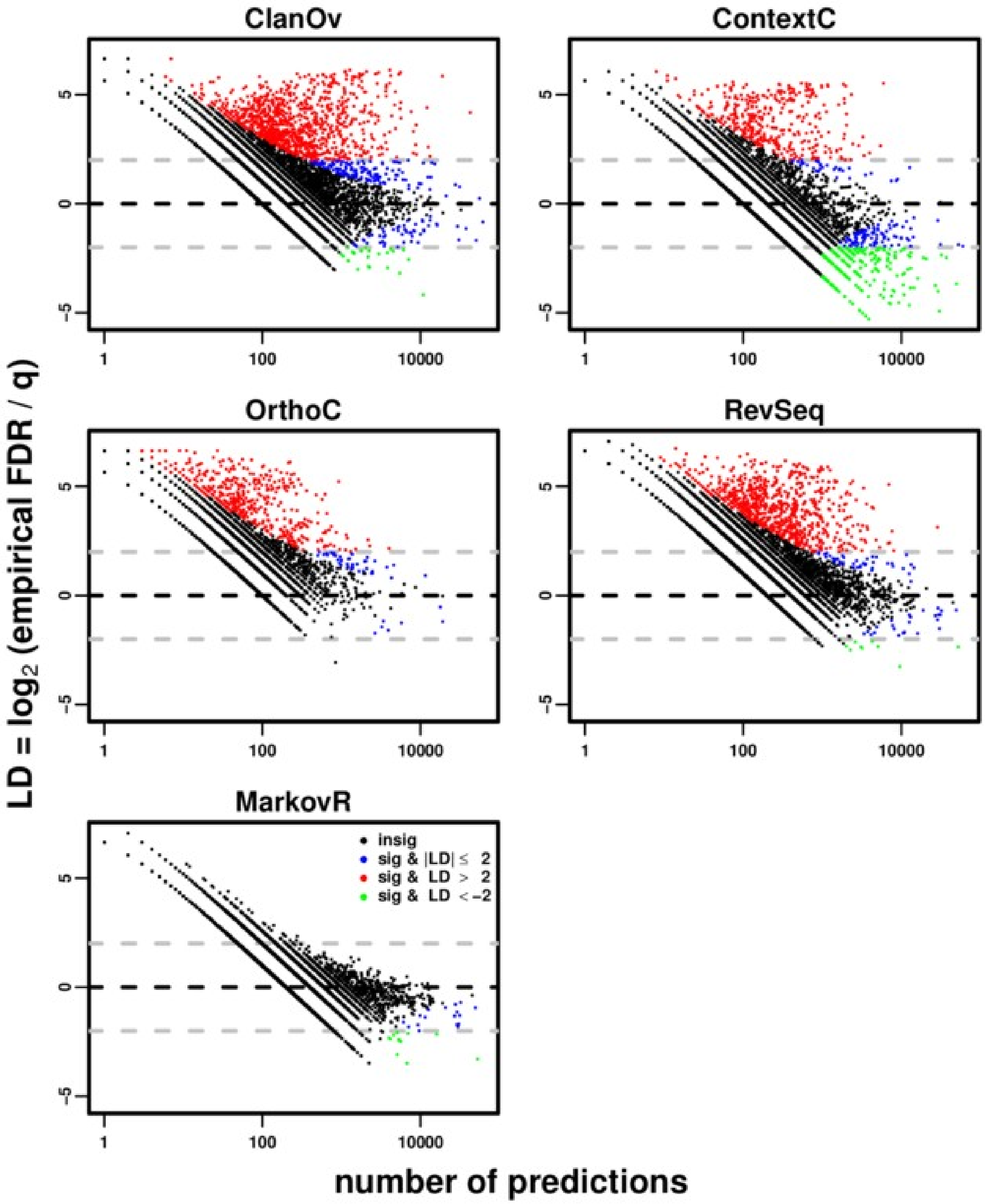

Figure I. Extended version of domain families with empirical FDRs that differ significantly from expectation. Extends **Fig 6** to include the OrthoC, RevSeq, and MarkovR tests. See the legend of **Fig 6** for further information.

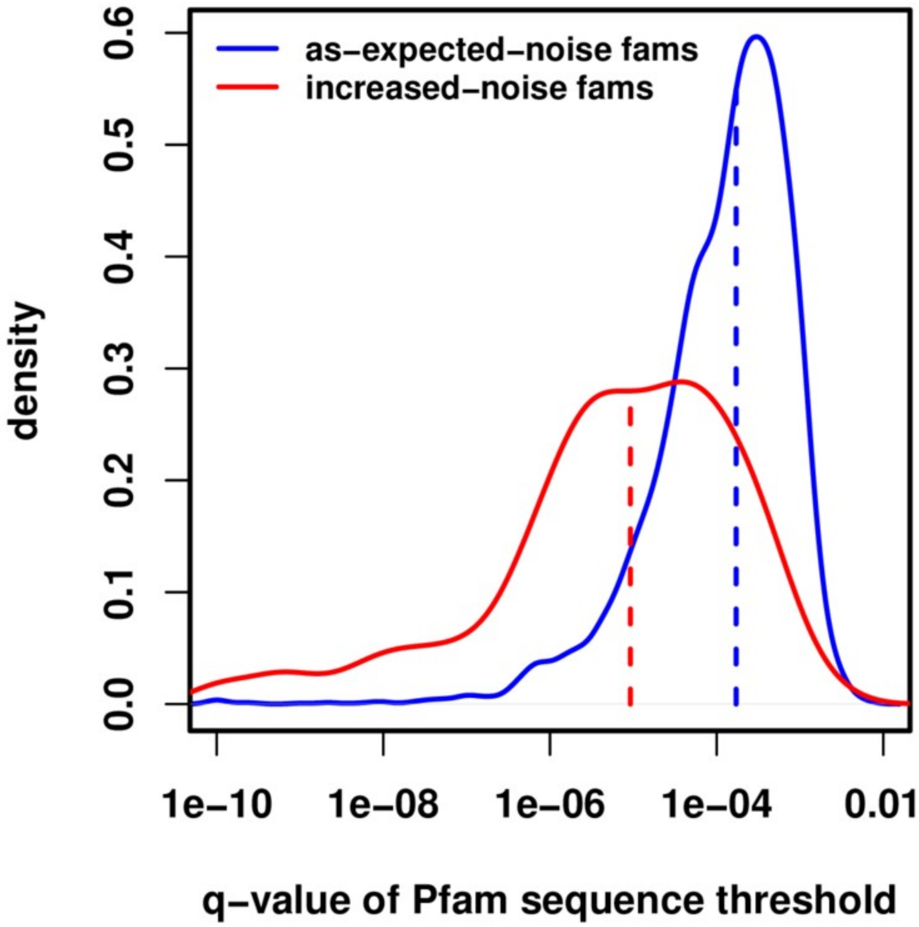

Figure J. Pfam thresholds somewhat compensate in increased-noise families. The *q*-value equivalent of each Pfam sequence threshold was computed by using the relationships between bitscores, *p*-values and *q*-values observed on UniRef50. The blue and red *q*-value distributions (the density is of $\log_{10} q$) correspond to the "as-expected-noise" and "increased-noise" subsets of Pfam families, respectively (see **Results**). The dashed lines correspond to the medians of the distributions with matching colors. For visualization purposes, *q*-values smaller than 1e-10 were displayed as 1e-10.

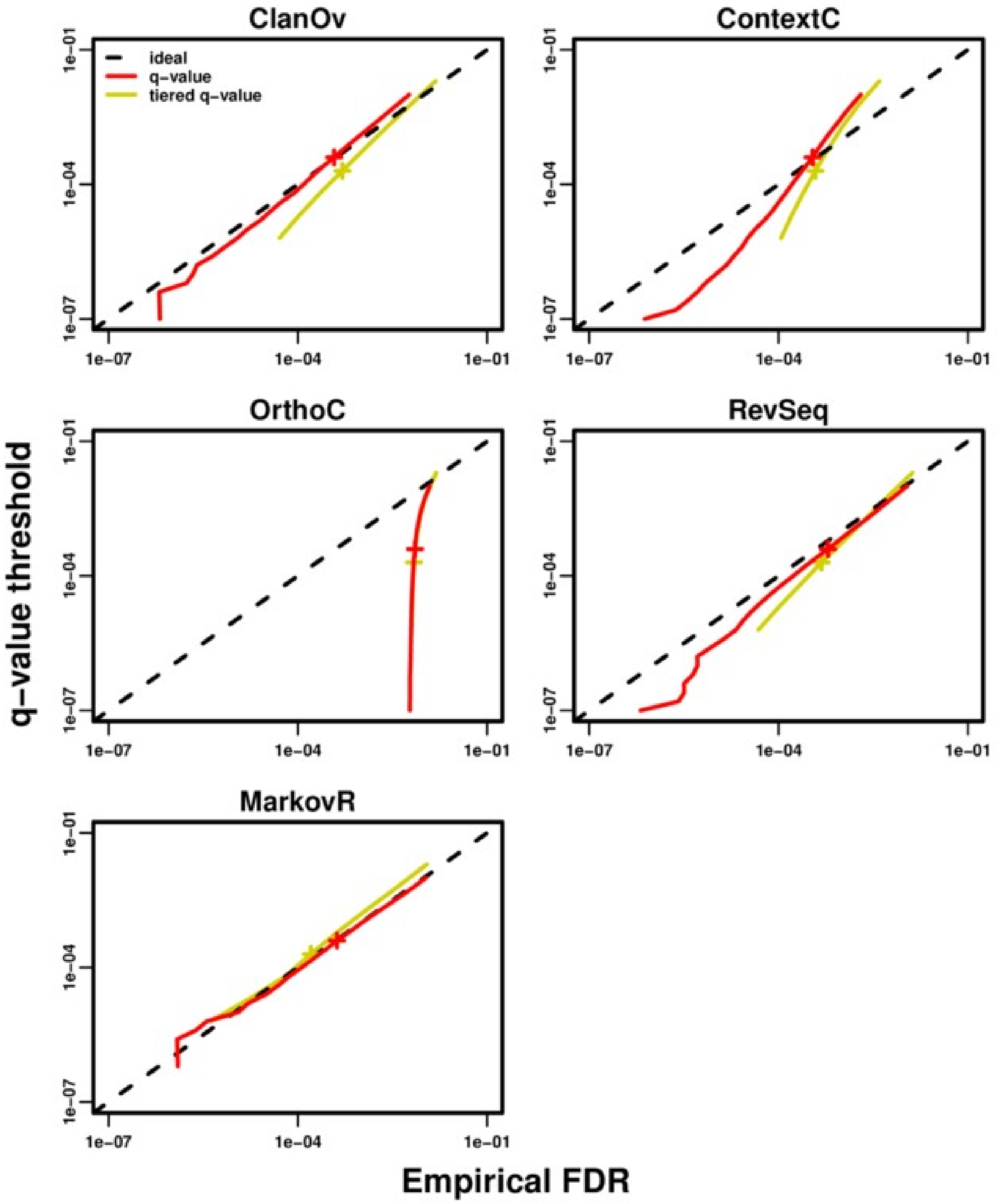

Figure K. Comparison of *q*-value thresholds and empirical FDRs, restricted to families with as-expected noise. Like **Fig H** except that only families with as-expected noise are used in the benchmarks. See **Fig 5** and **Fig H**.

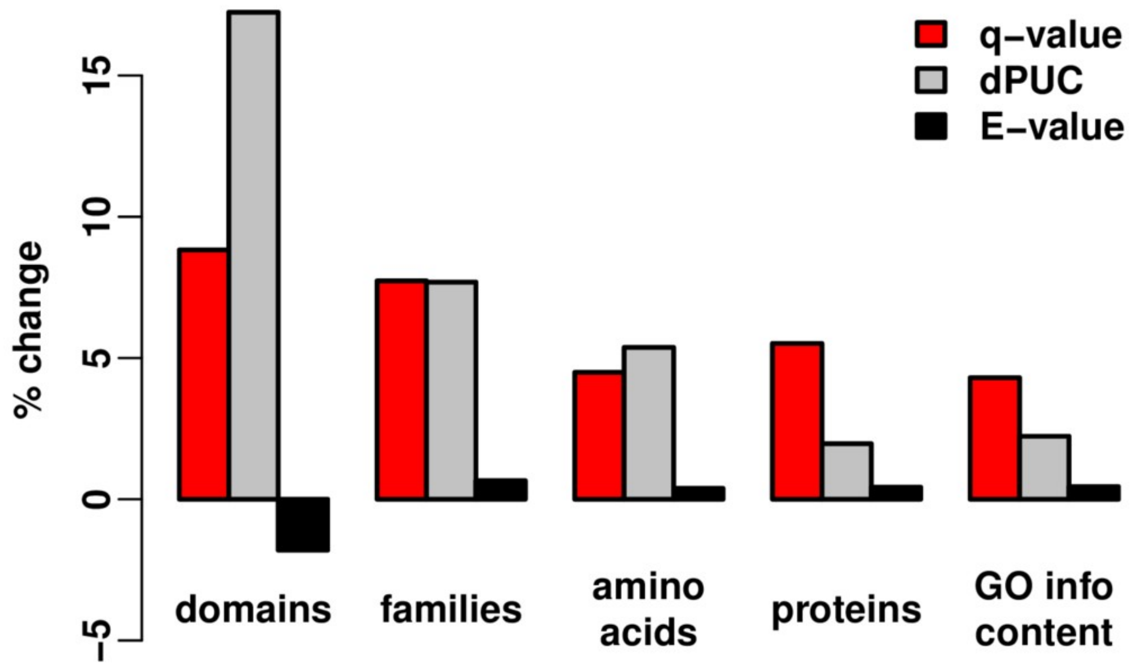

Figure L. Changes over many metrics for *q*-values and dPUC relative to the Standard Pfam, restricted to families with as-expected noise. Like **Fig 4** except that only families with as-expected noise, rather than all families, are used in the benchmarks.

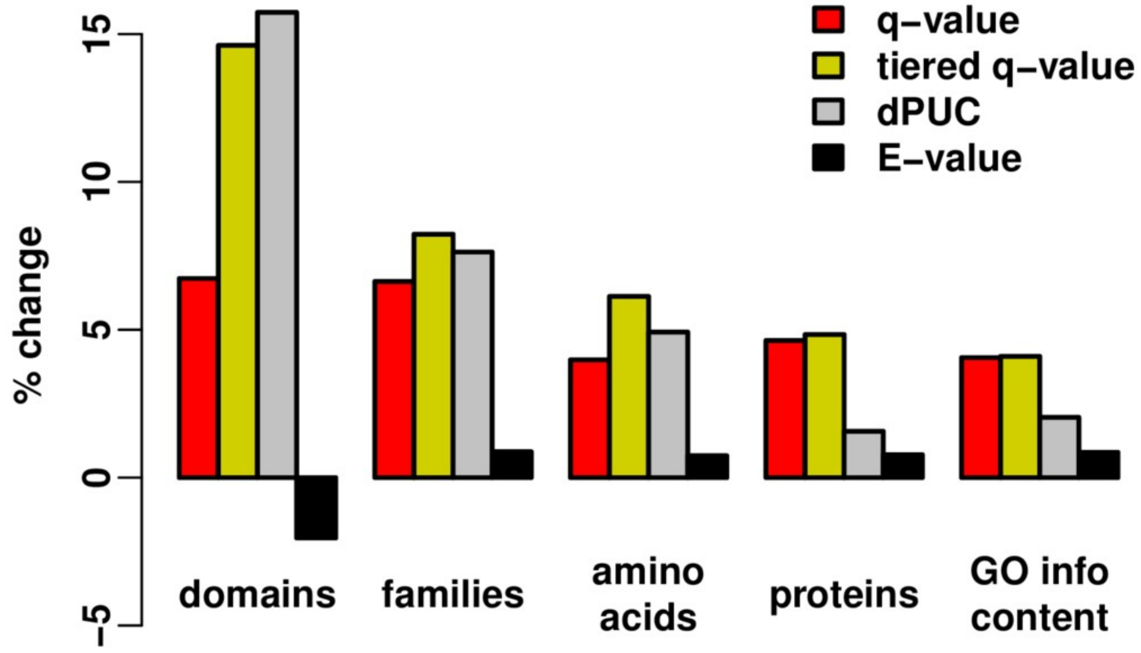

Figure M. Changes over many metrics for tiered *q*-values relative to the Standard Pfam. Extends **Fig 4** to include tiered *q*-values at a per-tier threshold of 1e-4. See **Fig 4**.

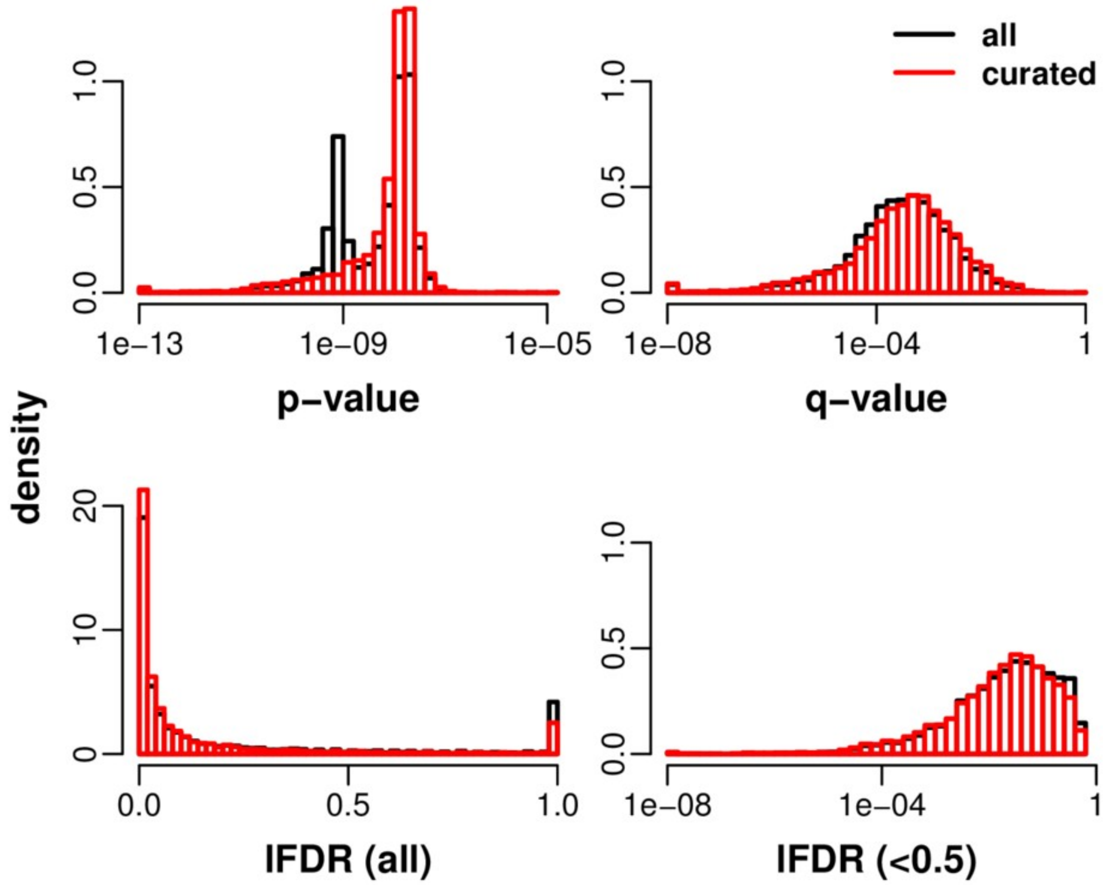

Figure N. Standard Pfam sequence thresholds mapped onto *p*-values, stratified *q*-values and lFDRs. We transformed bitscores to *p*-values, stratified *q*-values and lFDRs as computed on UniRef50. Black histograms includes all Pfam families, while red "curated" excludes families with Pfam thresholds of 25 and 27 bits. Full lFDR histograms (note linear *x*-axis, other panels have log scales) contain values larger than 0.5 due to an artifact we are unable to rectify (**Supp. Results**), so the second histogram only includes lFDRs < 0.5 to find a stratified threshold that Pfam curators are comfortable with. Densities for panels with log scales are $\log_{10}$ of each statistic. Histograms were truncated such that every *p*-value <1e-13 was set to that value, and similarly for *q*-values and lFDRs <1e-8.
